# Quantification of the risk of liver injury associated with flucloxacillin: a UK population-based cohort study

**DOI:** 10.1093/jac/dkx183

**Published:** 2017-07-01

**Authors:** Kevin Wing, Krishnan Bhaskaran, Louise Pealing, Adrian Root, Liam Smeeth, Tjeerd P. van Staa, Olaf H. Klungel, Robert F. Reynolds, Ian Douglas

**Affiliations:** 1 Department of Non-communicable Disease Epidemiology, Faculty of Epidemiology and Population Health, London School of Hygiene and Tropical Medicine, London, UK; 2 Nuffield Department of Primary Care Health Sciences, University of Oxford, Oxford, UK; 3 Health eResearch Centre, University of Manchester, Manchester, UK; 4 Department of Pharmacoepidemiology, Utrecht Institute for Pharmaceutical Sciences (UIPS), Utrecht University, Utrecht, The Netherlands; 5 Epidemiology, Pfizer, New York, NY, USA

## Abstract

**Background:**

Flucloxacillin is an established cause of liver injury. Despite this, there are a lack of published data on both the strength of association after adjusting for potential confounders, and the absolute incidence among different subgroups of patients.

**Objectives:**

To assess the relative and absolute risks of liver injury following exposure to flucloxacillin and identify subgroups at potentially increased risk.

**Methods:**

A cohort study between 1 January 2000 and 1 January 2012 using the UK Clinical Practice Research Datalink, including 1 046 699 people with a first prescription for flucloxacillin (861 962) or oxytetracycline (184 737). Absolute risks of experiencing both symptom-defined (jaundice) and laboratory-confirmed liver injury within 1–45 and 46–90 days of antibiotic initiation were estimated. Multivariable logistic regression was used to estimate 1–45 day relative effects.

**Results:**

There were 183 symptom-defined cases (160 prescribed flucloxacillin) and 108 laboratory-confirmed cases (102 flucloxacillin). The 1–45 day adjusted risk ratio for laboratory-confirmed injury was 5.22 (95% CI 1.64–16.62) comparing flucloxacillin with oxytetracycline use. The 1–45 day risk of laboratory-confirmed liver injury was 8.47 per 100 000 people prescribed flucloxacillin (95% CI 6.64–10.65). People who received consecutive flucloxacillin prescriptions had a 1–45 day risk of jaundice of 39.00 per 100 000 (95% CI 26.85–54.77), while those aged >70 receiving consecutive prescriptions had a risk of 110.57 per 100 000 (95% CI 70.86–164.48).

**Conclusions:**

The short-term risk of laboratory-confirmed liver injury was >5-fold higher after a flucloxacillin prescription than an oxytetracycline prescription. The risk of flucloxacillin-induced liver injury is particularly high within those aged >70 and those who receive multiple flucloxacillin prescriptions. The stratified risk estimates from this study could help guide clinical care.

## Introduction

Flucloxacillin is an antibiotic of the penicillin class that has a broad range of uses in the treatment of Gram-positive bacterial infections, including skin and soft tissue infections, respiratory tract infections, urinary tract infections, meningitis and prophylaxis during surgery.^1^ First available in 1960, case reports appeared in the 1980s of an adverse drug reaction in which the patient developed serious liver injury, which in some cases could be fatal.^2^ While commonly and increasingly prescribed in the UK,^3^ flucloxacillin is not marketed in the USA and some European countries, where alternative therapies perceived to have a better safety profile are used (such as dicloxacillin).

Previous work has shown flucloxacillin to be associated with liver injury at a frequency of ∼8 per 100 000 people exposed within the general population.^4–6^ Liver injury may occur up to 45 days from initiation of treatment, can be prolonged and is characterized by a predominantly cholestatic pattern of liver test results, and symptoms including jaundice. A number of epidemiological studies have identified an association with increased age, prolonged duration of use and female gender identified as possible risk factors.^6–8^ Despite this, there are a lack of available data either in the literature or prescribing information on (i) the strength of association after adjusting for potential confounders, or (ii) the absolute risk of either laboratory-confirmed or symptom-defined liver injury associated with flucloxacillin within these potentially high-risk groups.

The aims of this study were (i) to measure the association between being prescribed flucloxacillin and liver injury (compared with being prescribed oxytetracycline) after adjusting for potential confounders of the association, and (ii) to quantify the risk of both symptom-defined (jaundice) and laboratory-confirmed injury within both the general population and subgroups at potentially increased risk.

## Materials and methods

### Study design

The study design was a cohort analysis of the association between flucloxacillin and liver injury, with oxytetracycline as a comparator drug. Oxytetracycline was selected as it is an antibiotic that is not considered hepatotoxic and that, in the clinical context within which the study was set, is used for a number of the same conditions as flucloxacillin, including skin infections, respiratory tract infections and urinary tract infections (see Supplementary data section 1, available at *JAC* Online).

### Setting

The study was performed within the UK Clinical Practice Research Datalink (CPRD), which contains comprehensive anonymized diagnostic, prescribing and lifestyle records on patients from >625 NHS primary care practices from across the UK (∼12 million total patients, broadly representative of the UK population).^9^ Further information is provided in the Supplementary data (section 1) and elsewhere.^9^

### Participants

The cohort was selected from patients actively registered in the CPRD between 1 January 2000 and 1 January 2012. The exposed group was composed of people aged >18 years with at least one prescription for flucloxacillin and at least 6 months of research-quality prescription history in CPRD prior to their first recorded prescription of flucloxacillin (see Supplementary data, section 1).

Patients with diseases or conditions that were likely to cause liver-related symptoms in their CPRD record within 6 months prior to their first recorded flucloxacillin prescription were excluded (see Supplementary data section 2), as were people with any liver test results that met the criteria for drug-induced liver injury^10^ (DILI; Table 1) within the previous 6 months. Women who were pregnant at the time of their first recorded flucloxacillin prescription were also excluded (to avoid liver symptoms caused by cholestasis in pregnancy).

**Table 1.** Classification of DILI based on liver test results^10^

Type of liver injury	Liver test result
Characteristic of any DILI	ALT ≥5× ULN or
	ALP ≥2× ULN or
	ALT ≥3× ULN and Bil >2× ULN
Characteristic of hepatocellular type of DILI	R^a^ ≥5
Characteristic of mixed type of DILI (=cholestatic hepatitis)	R > 2 and <5
Characteristic of pure cholestatic type of DILI	R ≤ 2

ALP, alkaline phosphatase; Bil, bilirubin; ULN, upper limit of normal.

a R = (ALT/ULN)/(ALP/ULN).

People prescribed oxytetracycline were selected as the comparator group, as oxytetracycline is an antibiotic with a similar range of indications to flucloxacillin that is not considered to cause liver injury.^6^ The exclusion criteria applied to the oxytetracycline group were the same as in the group exposed to flucloxacillin.

### Ethics

Ethics approval was obtained from the Clinical Practice Research Datalink Independent Scientific Advisory Committee (approval number 12_049) and the LSHTM Research Ethics Committee (approval number 6215).

### Exposures, outcomes and covariates

#### Exposures

Exposures were determined from CPRD prescription records. Based on results from previous studies suggesting injury may occur within a period of 6 weeks after flucloxacillin initiation,^5^^,^^6^ a person was considered exposed and at risk for 45 days after the start of a first prescription for flucloxacillin or oxytetracycline. The date of the first prescription was the index date, and people receiving both drugs on the index date were included in the flucloxacillin group only. Anyone who received oxytetracycline on their index date but then received flucloxacillin within 45 days was reassigned to the flucloxacillin group, and their index date updated appropriately. A categorical *number of flucloxacillin prescriptions* variable was created, which recorded how many prescriptions for flucloxacillin an individual received between their index date and the earliest of: an outcome event, exclusion event, transfer out of the database, death or day 45. For those in the exposed to flucloxacillin group, a (comparator) day 46–90 exposure period was also included for analysis.

#### Outcomes

Diagnostic terms, code lists and laboratory parameters for the outcome were selected based upon a review of 12 studies^6^^,^^11–21^ identified by a systematic literature review performed for a previous study on liver injury.^22^ Final review of outcome definitions was performed by a member of the study team who is a General Practitioner and Professor in Clinical Epidemiology (L. S.), and a list of final terms is provided in the Supplementary data (section 3).

**Figure 1.**
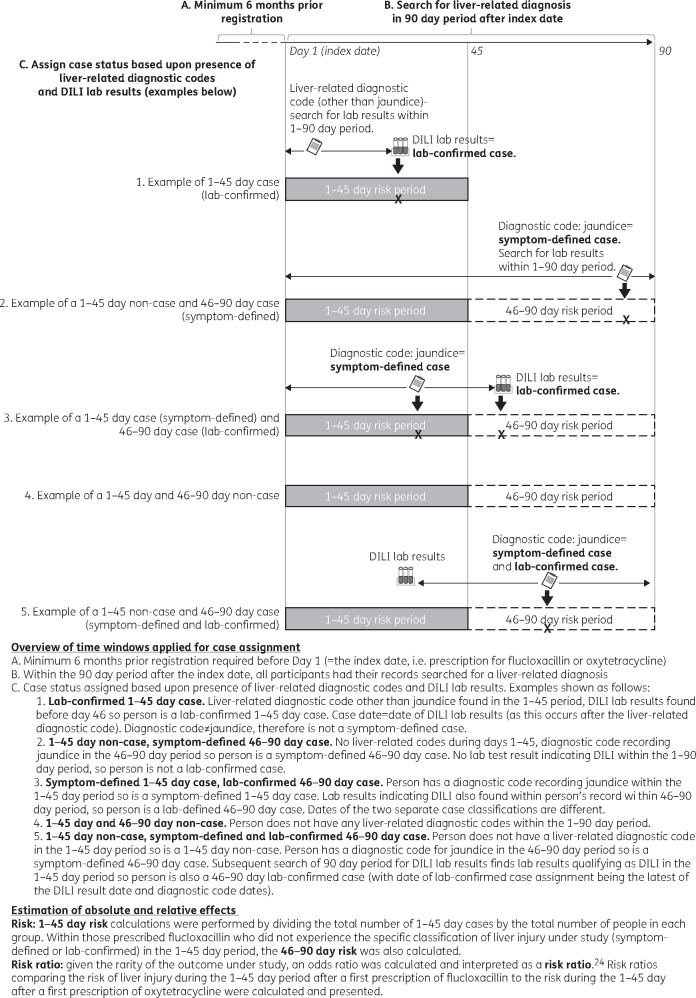
Overview of time windows used for case assignment and analysis performed for the exposed and comparator groups of the flucloxacillin and liver injury cohort study.

Assignment of outcome status was performed blinded to drug exposure status. Initially, potential cases were selected as people with any of a relatively broad list of liver-related diagnoses (Supplementary data section 3) within the 90 day period after their index date (Figure 1). The 1–90 day period was searched (rather than just the 1–45 day risk period) because for those prescribed flucloxacillin, we wanted to compare the risk of injury in the 46–90 day period with that of the 1–45 day period. Any liver test results for bilirubin, alkaline phosphatase (ALP) and ALT recorded within the 1–90 day period were then identified for these potential cases. Blood levels of these enzymes taken from the same blood sample are standard parameters for indicating and classifying DILI based upon the R value (a ratio of ALT to ALP, detailed in Table 1). Data management was performed to obtain R values as detailed previously.^22^

The R values and Read codes were then used to define the following two potential liver injury case statuses:

1. **Symptom-defined case**: people who had a liver-related diagnosis code within the 90 day period following the index date for any jaundice-related diagnosis or symptom (see Supplementary data section 3)
2. **Laboratory-confirmed case**: people who had both of the following within the 90 day period following the index date: (i) any of the liver-related diagnoses detailed in Supplementary data section 3, and (ii) a liver test result indicative of DILI (Table 1).

A symptom-only (jaundice) case definition was included due to the unavailability of laboratory test results from secondary care within CPRD, meaning that reliance on only laboratory test results to define cases may under-ascertain the number of cases.^22^

The case-date for final symptom-defined cases was the date of jaundice, while for final laboratory-confirmed cases, it was the latest of the liver-related diagnosis or laboratory test result indicating DILI (Figure 1). The full electronic health record of all potential cases for the period from 6 months prior to the index date up until the case date was then reviewed by a clinician (A. R.), blinded to drug exposure status. Potential cases without any more likely causes of liver injury were designated as cases, while those with a more likely cause or liver-related symptoms occurring prior to the index date were considered exclusions, and either excluded from the analysis completely (if the exclusion event was prior to their index date) or were kept in the analysis but designated as non-cases (if the exclusion event happened after their index date but prior to their case date).

To assess the performance of our case detection method against an established method for assessing causality of DILI, we applied the RUCAM/CIOMS causality assessment method^23^ to each of the laboratory-confirmed cases (see Supplementary data section 3b).

#### Covariates and risk factors

Results of previous studies and a causal diagram were used to assist with the selection of covariates for the causal analysis. Age, gender, smoking, ethnicity, BMI, alcohol intake, socio-economic status, use of other drugs known to cause liver injury and calendar period were all included as potential measurable confounders. Further details are provided in Supplementary data section 4a.

Potential risk factors for increased susceptibility to flucloxacillin-induced liver injury were selected based on the results of previous studies^6–8^ and included gender, age and number of prescriptions.

### Statistical analysis

#### Descriptive analysis

Covariates were tabulated by exposure status, before the number of cases within the 1–90 day period within each drug-exposure group was calculated. For the flucloxacillin group, the proportion of type of liver injury (hepatocellular versus cholestatic), characteristic symptoms of cases and median time from first prescription until case assignment were also tabulated.

#### Overall risk of liver injury

The 1–45 day risk of liver injury for each drug was calculated by dividing the total number of events within the 45 day period after the index date by the number of patients in each exposure group (Figure 1). Ninety-five percent CIs were calculated based on the Poisson distribution of injury events within each exposure group and the risk of liver injury occurring per 100 000 people within each of the exposure groups was tabulated. The risk of liver injury in the 46–90 day period after exposure to flucloxacillin was also calculated (Figure 1).

#### Association between flucloxacillin and liver injury

For the analysis of the association between flucloxacillin and liver injury, all relative effects were calculated as odds ratios, which given the rarity of the outcomes under study were interpreted as risk ratios (RRs)^24^ (and will be referred to as such subsequently in this article).

Crude RRs comparing the risk of liver injury during the 1–45 day period after a first prescription of flucloxacillin to the risk during the 1–45 day after a first prescription of oxytetracycline (Figure 1) were obtained. A logistic regression model was then constructed, with potential confounders included as informed by the causal diagram, to estimate an overall adjusted RR for the effect of flucloxacillin on liver injury.

#### Analysis of risk factors for flucloxacillin-induced liver injury

Risks per 100 000 people exposed to flucloxacillin and multivariable adjusted RRs were calculated and tabulated across all categories of each potential risk factor, with tests-for-trend applied where appropriate. Graphs were plotted to illustrate the change in risk across categories for potential risk factors shown to increase susceptibility to injury.

#### Missing data and sensitivity analyses

A description of the handling of missing data is provided in the Supplementary data (section 4b).

The following sensitivity analyses were performed: (i) removing those on co-fluampicil; (ii) removing those in the heaviest drinking category; (iii) removing people prescribed both flucloxacillin and oxytetracycline; and (iv) considering people with exclusion codes between drug prescription and an outcome event as cases.

All analysis was performed using STATA (StataCorp LP, version 14.0).

## Results

### Participants

**Figure 2.**
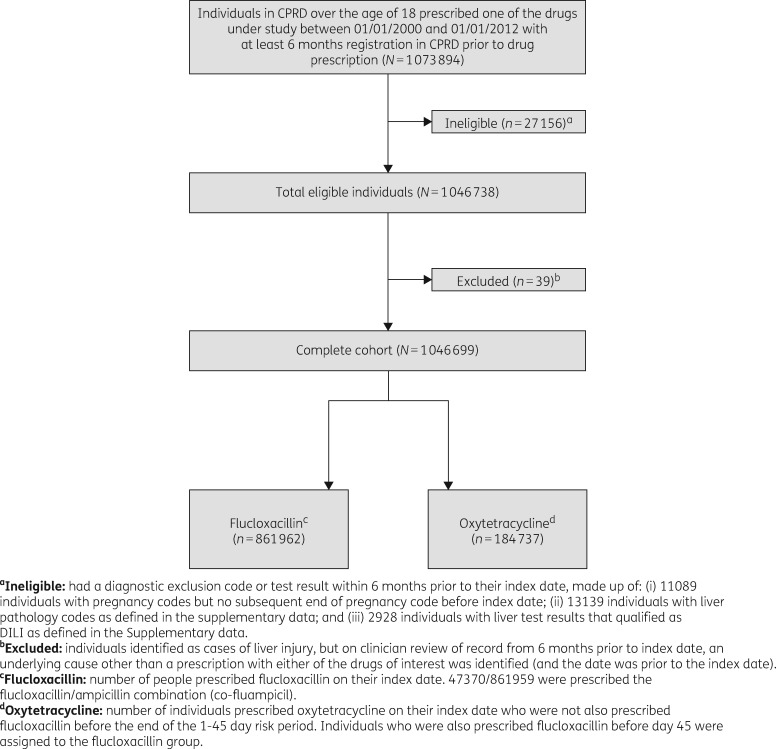
Flow of number of individuals included in the cohort study of the association between flucloxacillin (compared with oxytetracycline) and liver injury.

Between 1 January 2000 and 1 January 2012, 1 073 894 people aged 18 years and over were identified in CPRD who received a first prescription for either flucloxacillin or oxytetracycline and had been registered in the database for at least 6 months (Figure 2). The removal of 27156 people who did not meet the necessary eligibility criteria left 1046738 people in the cohort. An additional 39 were found to have reasons for exclusion during a detailed potential case review, leaving a final cohort of 1 046 699 people for analysis.

### Descriptive data

Background characteristics of participants are shown in Table 2. There were 861 962 people prescribed flucloxacillin and 184 737 prescribed oxytetracycline. Of those prescribed oxytetracycline, 56% were female, compared with 54% of those prescribed flucloxacillin, and a higher proportion of those in the oxytetracycline group (55%) had an index date prior to 2006 than in the flucloxacillin group (48%). Oxytetracycline patients included a higher proportion of people on other drugs likely to cause liver injury than flucloxacillin patients (81% versus 52%). There was no difference in recorded ethnicity between the groups, and minimal differences in the distribution of all other characteristics between exposure groups. Ethnicity data were missing for 37% of the cohort.

**Table 2.** Characteristics of participants included in the cohort analysis of the association between flucloxacillin (compared with oxytetracycline) and liver injury, by exposure status

Variable	Oxytetracycline (*N* = 184 737)	Flucloxacillin (*N* = 861 962)
Age at index date, years, median (IQR)	50 (35–65)	48 (34–65)
Gender		
male	81 316 (44)	394 125 (46)
female	103 421 (56)	467 834 (54)
Date of index prescription		
2000–01	32 439 (17)	112 188 (13)
2002–03	34 830 (19)	143 752 (17)
2004–05	32 615 (18)	156 808 (18)
2006–07	30 090 (16)	159 304 (18)
2008–09	29 217 (16)	153 679 (18)
2010–11	25 546 (14)	136 228 (16)
Prescriptions for other causes of liver injury^a^		
none	34 529 (19)	415 687 (48)
less common cause	143 164 (77)	399 846 (47)
more common cause	7044 (4)	46 426 (5)
Smoking status		
non-smoker	84 864 (46)	382 320 (44)
ex-smoker	40 979 (22)	219 122 (25)
current smoker	55 343 (30)	242 314 (29)
missing	3551 (2)	18 203 (2)
BMI		
<20	10 923 (6)	48 451 (6)
20–25	55 689 (30)	247 583 (29)
>25	95 215 (52)	447 203 (52)
missing	22 910 (12)	118 722 (13)
Alcohol intake		
non-drinker	20 831 (11)	97 065 (11)
ex-drinker	5581 (3)	28 277 (3)
current NOS	5852 (3)	27 452 (3)
≤2 units/day	30 424 (16)	139 300 (16)
3–6 units/day	84 057 (46)	381 539 (44)
>6 units/day	13 232 (7)	66 576 (8)
missing	24 760 (14)	121 750 (15)
Socio-economic status (SES)^b^		
1 (highest SES)	33 239 (18)	153 552 (18)
2	29 919 (16)	145 586 (17)
3	27 753 (15)	140 223 (16)
4	27 541 (15)	131 425 (15)
5 (lowest SES)	19 122 (10)	102 723 (12)
missing	47 163 (26)	188 450 (22)
Ethnicity^c^		
white	93 400 (51)	440 740 (51)
South Asian	3010 (2)	14 487 (2)
black	1445 (1)	8566 (1)
other	1470 (1)	6202 (1)
mixed	392 (0)	2238 (0)
not stated	14 390 (8)	70 946 (8)
missing	70 630 (37)	318 780 (37)

Data are presented as *n* (%) unless otherwise indicated.

a Prescription counted if it occurred anytime from 1 month prior to index date or between index and before end of follow-up. Less or more common in relation to flucloxacillin, as reported in the literature.

b Linked data, only available for practices in England, based on index of Multiple Deprivation (individual patient postcode) or otherwise practice level score based upon practice postcode (if no individual-level data).

c Obtained from CPRD, unless none found, in which case from HES if patient from a linked practice.

### Description of liver injury cases

Within 90 days from the index prescription, there were 183 symptom-defined cases (169 in the exposed to flucloxacillin group) and 108 laboratory-confirmed cases (102 in the exposed to flucloxacillin group). The type of liver injury within cases exposed to flucloxacillin was primarily (pure or mixed) cholestatic (69% of cases), and the median time from first flucloxacillin prescription until symptom-defined case assignment was 38 days (IQR 27–47), increasing to 40 days (IQR 32–48) for laboratory-confirmed cases (Supplementary data section 5, Table S1).

#### Risk of liver injury associated with flucloxacillin

Table 3 shows absolute risk figures and both crude and multivariable adjusted results of the association between flucloxacillin and liver injury (compared with oxytetracycline).

**Table 3.** One to 45 day risk of liver injury by exposure to flucloxacillin or oxytetracycline and crude and multivariable adjusted risk ratios (RRs) (comparing the flucloxacillin 1–45 day period with the oxytetracycline 1–45 day period)

Case definition^a^/ exposure group	No. with outcome	Patients	45 day risk (95% CI) (per 100 000 patients prescribed the drug)	Crude RR (95% CI)	Multivariable RR^b^ (95% CI)
Symptom-based only					
oxytetracycline	7	184 737	3.79 (1.52–7.81)	1	1
flucloxacillin	122	861 962	14.15 (11.75–16.92)	3.74 (1.74–8.00)	3.73 (1.73–8.03)
Laboratory-confirmed					
oxytetracycline	<5^3^	184 737	1.62 (3.35–4.75)	1	1
flucloxacillin	73	861 962	8.47 (6.64–10.65)	5.22 (1.65–16.57)	5.22 (1.64–16.62)

a Symptom-based only: diagnostic code for jaundice present within the 45 day risk period being analysed. Laboratory-confirmed: both of the following present within the 45 day risk period being analysed: (i) any of the diagnostic codes listed in Supplementary data section 3, and (ii) liver test results indicating DILI (according to Aithal *et al.*^10^). Both definitions: all other more likely causes of the liver symptoms ruled out by clinician review of full electronic health record in the 6 month period before the case date.

b Adjusted for age, gender, date of index prescription, prescriptions for other drugs likely to cause liver injury, smoking status, BMI, alcohol intake, socio-economic status and ethnicity. Missing covariate data taken account of using multiple imputation by chained equations, with all available variables included in the multiple imputation model.

There were 73 of 861 962 people prescribed flucloxacillin with laboratory-confirmed liver injury within the 45 days after prescription, giving a 1–45 day risk of flucloxacillin-induced liver injury of 8.47 cases per 100 000 people (95% CI 6.64–10.65). The risk of laboratory-confirmed injury for those exposed to oxytetracycline within the same period was 1.62 per 100 000 people (95% CI 3.35–4.75), while the risk within those in the flucloxacillin group within the 46–90 day period from first prescription was 3.45 per 100 000 (95% CI 2.31–4.95) (data not shown). For the case definition requiring only a symptom or diagnosis of jaundice (symptom-defined), the risk of liver injury within the 1–45 day period for those prescribed flucloxacillin was almost double that of the laboratory-confirmed case definition (14.15 per 100 000, 95% CI 11.75–16.92) (Table 3).

The crude RR for the association between flucloxacillin and laboratory-confirmed liver injury was 5.22 (95% CI 1.65–16.57). There was no change in this estimate following multivariable adjustments (RR 5.22, 95% CI 1.64–16.62). The multivariable RR for the symptom-defined outcome was lower than the laboratory-confirmed estimate, but had narrower CIs (RR 3.73, 95% CI 1.73–8.03).

### Risk factors for flucloxacillin-induced liver injury

There was strong evidence that increasing age was a risk factor for flucloxacillin-induced liver injury (*P* test-for-trend < 0.001 for both symptom-based and laboratory-confirmed outcomes), with a marked increase in the 1–45 day risk of injury in those aged >70 (e.g. multivariable-adjusted RR for laboratory-confirmed liver injury comparing those in the 70–79 year old age group with those aged 18–49: 23.26, 95% CI 7.88–68.67) (Table 4). There was also strong evidence for an increased 1–45 day risk of injury with increasing number of prescriptions (*P* test-for-trend < 0.001), with people receiving three or more prescriptions within the 1–45 day risk period experiencing 9.37 times the 1–45 day risk of laboratory-confirmed injury (95% CI 4.40–19.95) than those receiving a single prescription within this period, after adjusting for age, gender and concomitant prescriptions for other causes of liver injury. For gender, there was a suggestion across both outcomes that females had a slightly increased risk of injury, although the 95% CI did not rule out a decreased risk (e.g. multivariable RR for symptom-based injury comparing females to males: 1.43, 95% CI 0.98–2.08).

**Table 4.** Risks and multivariable adjusted risk ratios (RRs) for liver injury within those exposed to flucloxacillin (for the 1–45 day period after exposure) for laboratory and symptom-based cases by potential risk factors age, gender and number of prescriptions

Case definition^a^	Risk factor	No. with outcome	Patients	Risk^b^ (95% CI)	Multivariable RR^c^ (95% CI)
Symptom-based only (*n* = 122)	Age				
	18–49	13	453 636	2.87 (1.53–4.90)	1^6^
	50–59	19	129 179	14.71 (8.86–22.97)	5.02 (2.47–10.19)
	60–69	14	111 368	12.57 (6.87–21.09)	4.18 (1.95–8.99)
	70–79	41	91 443	44.84 (32.18–60.82)	14.31 (7.51–27.26)
	80+	35	76 336	45.85 (31.94–63.76)	13.87 (7.16–26.86)
	Gender				
	male	43	394 126	10.91 (7.90–14.70)	1
	female	79	467 836	16.89 (13.37–21.04)	1.43 (0.98–2.08)
	No. of prescrs				
	1	88	777 353	11.45 (9.19–14.09)	1^d^
	2	26	74 431	33.59 (21.74–49.58)	2.45 (1.57–3.82)
	3+	8	10 178	78.60 (33.94–154.82)	5.06 (2.44–10.46)
Laboratory-confirmed (*n* = 73)	Age				
	18–49	4	453 636	0.89 (0.24–2.26)	1^d^
	50–59	13	129 179	10.06 (5.36–17.21)	10.79 (3.50–33.19)
	60–69	10	111 368	8.97 (4.31–16.51)	8.83 (2.74–28.50)
	70–79	23	91 443	25.15 (15.95–37.74)	23.26 (7.88–68.67)
	80+	23	76 336	30.13 (19.10–45.21)	25.42 (8.58–75.33)
	Gender				
	male	24	394 126	6.09 (3.90–9.06)	1
	female	49	467 836	10.47 (7.75–13.85)	1.61 (0.98–2.65)
	No. of prescrs				
	1	46	777 353	5.92 (4.33–7.89)	1^d^
	2	19	74 431	25.53 (15.37–39.86)	3.50 (2.05–6.00)
	3+	8	10 178	78.60 (33.94–154.82)	9.37 (4.40–19.95)

prescrs, prescriptions.

a Symptom-based only: diagnostic code for jaundice present within 1–45 day risk period. Laboratory-confirmed: both of the following present within the 1–45 day risk period: (i) any of the diagnostic codes listed in Supplementary data section 3, and (ii) liver test results indicating DILI (according to Aithal *et al.*^10^). Both definitions: all other more likely causes of the liver symptoms ruled out by clinician review of full electronic health record in the 6 month period before the case date.

b Per 100 000 people prescribed flucloxacillin.

c Adjusted for date of index prescription, concomitant therapies for drugs considered causes of liver injury and all other variables in this table.

d *P* (test for trend) < 0.001.

**Figure 3.**
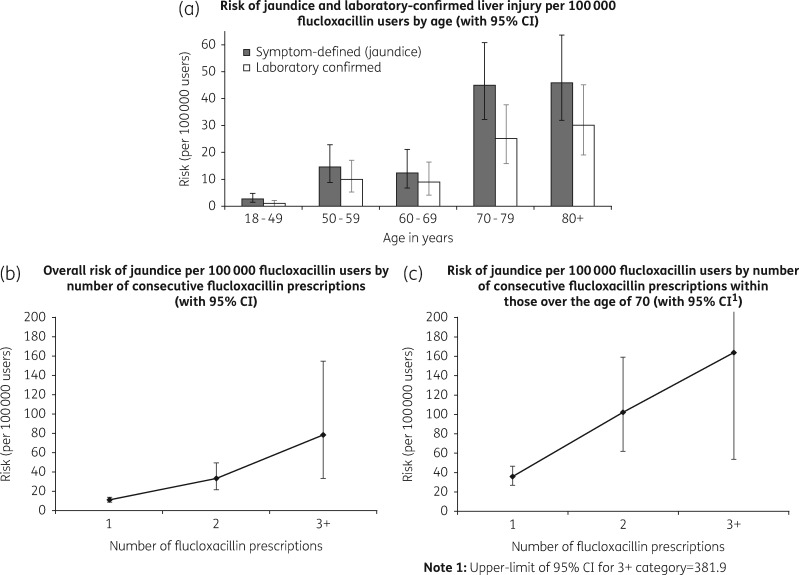
Illustration of change in absolute risk of flucloxacillin-induced liver injury by (a) increasing age (for both jaundice and laboratory-confirmed outcomes) and (b,c) increasing number of prescriptions [for jaundice, showing (b) overall risk and (c) risk within those aged >70].

Considering the absolute 1–45 day risk per 100 000 people exposed to flucloxacillin, the risk of jaundice in the 18–49 year old age group was 2.87 (95% CI 1.53–4.90), increasing to 14.71 (95% CI 8.86–22.97) in the 50–59 year old age group (Table 4 and Figure 3a). Within those aged >70 years, the absolute risk of jaundice was 45.30 per 100 000 people (95% CI 35.69–56.69). In the overall population the risk of jaundice for those receiving a single prescription was 11.45 (95% CI 9.19–14.09), increasing to 78.60 per 100 000 (95% CI 33.94–154.82) within people receiving three or more flucloxacillin prescriptions (Table 4 and Figure 3b and c). People >70 years receiving three or more prescriptions had a risk of jaundice of 163.83 (95% CI 53.21–381.9) (Figure 3b and c), while the >70 year olds receiving two or more had a risk of 110.57 per 100 000 (95% CI 66.35–154.79). Risk figures for laboratory-confirmed injury were generally smaller in magnitude but demonstrated similar changes by age group and increasing number of prescriptions (Table 4 and Figure 3a).

### Performance of case definition compared with the RUCAM/CIOMS method

The RUCAM/CIOMS method^23^ classified 63 of 73 (86%) of laboratory-confirmed cases from this study as ‘Probable (flucloxacillin) adverse drug reaction (ADR)’ (see Supplementary data section 3b for description of categories). The remaining 10 of 73 (14%) were classified as ‘Possible (flucloxacillin) ADR’. Of these, five were under the RUCAM/CIOMS age risk factor cut-off of 55 years old, with the remaining five having a prescription record for another drug that may have been a more likely cause of the observed injury. Within 1–45 day laboratory-confirmed cases aged >70, 91% (42 of 46) were classified as RUCAM/CIOMS probable.

### Pattern of liver injury by age

We performed a *post hoc* analysis of the 73 people exposed to flucloxacillin with laboratory-confirmed liver injury to investigate whether the pattern of liver injury associated with flucloxacillin use varied by age group. Thirty-eight of the 46 people aged ≥70 years had a cholestatic type of injury (83%, 95% CI 71%–94%), compared with 15 of 27 aged <70 years (56%, 95% CI 35%–76%, Mann–Whitney test *P* = 0.01).

### Sensitivity analyses and missing data

None of the sensitivity analyses performed had anything other than a negligible impact on the results obtained. There was minimal difference between univariable analysis results obtained using complete records compared with the multiply imputed dataset (Supplementary data section 5, Tables S2 and S3).

## Discussion

In this study, we have shown flucloxacillin to be associated with 5.22 (95% CI 1.64–16.62) times the 1–45 day risk of laboratory-confirmed liver injury than oxytetracycline after multivariable adjustments, with an absolute 1–45 day risk of 8.47 (95% CI 6.64–10.65) per 100 000 people prescribed the drug for the first time. There was strong evidence that increasing age and number of prescriptions were associated with increased flucloxacillin-induced liver injury, with those >70 years who received at least one additional flucloxacillin prescription within 45 days of their initial prescription having a risk of jaundice of 110.57 per 100 000 people (95% CI 66.35–154.79).

### Comparison with previous studies

Our estimate of the overall risk of laboratory-confirmed liver injury is comparable with previously published risk estimates of 7.57 (95% CI 3.63–13.92)^7^ and 8.48 (95% CI 5.43–12.61).^6^ While previous studies have estimated the relative effect of age on risk to be between 18.61 (comparing >55s versus <30s)^8^ and 6.1 (comparing >60 versus <60),^6^ to our knowledge, our large study is the first to estimate absolute risk figures by age categories, and has shown that those >70 years of age experience the highest risk. We found a 9-fold increased risk in people given three or more flucloxacillin prescriptions compared with those given one prescription, which is also consistent with previous work showing that those with >14 consecutive days of use have 7.13 times the risk of injury than people using for less than this period (95% CI 2.90–17.58).^8^ The size of our study has allowed us to demonstrate a dose (prescription)–response effect, and show that those aged >70 who receive more than one prescription within the 1–45 day period have a particularly elevated risk.

### Implications and further work

Current flucloxacillin prescribing information relating to hepatic side effects^1^ states that (i) jaundice affects <1 in 10 000 people, and (ii) the drug should be used cautiously in people >50 years of age. Our results suggest that flucloxacillin causes jaundice at a frequency closer to 1 in 7000 people in the overall population, that prolonged use is likely to increase the risk further, and those >70 years have ∼15-fold higher risk than those <50 years. This is a particular concern when considering recent flucloxacillin prescribing trends showing that people >70 years have both the highest prescribing rates and largest increase in rates.^3^ We would therefore hope that these findings could help physicians gain a greater understanding of the nature of the risk involved with prescribing flucloxacillin, and exercise particular caution in prescribing long treatment courses to those >70 years. In a clinical setting, the choice may be between flucloxacillin and another drug with known adverse effects on the liver—the absolute risk figures provided in our study would help inform clinicians’ prescribing decisions in this situation.

In terms of a mechanism for an age-dependent increase in the risk of flucloxacillin-induced liver injury, it is plausible that impaired renal function in the elderly could increase drug concentrations.^25^ Not all drugs associated with liver injury demonstrate a similar age-dependent increase in risk, however,^26^ suggesting there may be an alternative mechanism. An increased use of concomitant hepatotoxins amongst the elderly has also been suggested as contributing to the observed increased risk,^26^ but in our study we adjusted for use of a large number of known hepatotoxins. We did observe that patients >70 years had a higher proportion of cholestatic (versus hepatocellular) injury than those <70 (consistent with previous studies on DILI),^27^^,^^28^ and we hope this could help inform studies on the mechanism of flucloxacillin-induced liver injury in the future.

We would also hope that our findings might help further development of a predictive genetic test and/or elucidation of mechanism via genetic association studies. Genetic analysis has demonstrated the HLA-B*5701 genotype to be a major determinant of DILI due to flucloxacillin.^29^ Despite this finding, subsequent consideration of clinical utility^30^ showed that (based on an overall population prevalence of 8.5 per 100 000) predictive genetic testing for the reaction would be unfeasible, as 13 513 people would need screening to prevent 1 case. Assuming that all of the cases of jaundice attributed to flucloxacillin in this study fulfil the criteria for DILI (which we consider a fair assumption, given how clear an indicator jaundice is of a serious liver problem), calculating the number needed to test within those >70 using the drug reduces this number to 2512 (see Supplementary data section 6). Although still likely to be prohibitively high, further elucidation of characteristics associated with increased risk may allow the number needed to test to be reduced further for specific groups in the future.

### Limitations

It is likely that older people will have more liver tests performed, meaning that ascertainment bias could have affected our results. We found comparable results for jaundice-defined cases, however, making this an unlikely explanation for our results. There is no specific Read code or term to allow a clinician to record a case of DILI within CPRD, meaning that there was an element of clinical uncertainty around assigning case status. We attempted to overcome this by using a detailed algorithm based upon a literature search of diagnostic terms, defined standards for laboratory test patterns indicative of DILI and applying multiple case definitions. We were also able to demonstrate that 86% of the cases of liver injury that we attributed to flucloxacillin would have been assigned as ‘Probable’ flucloxacillin-induced liver injury by the RUCAM/CIOMS causality assessment method (91% of those in the >70 year old age group). Improved coding and linkages with, e.g. liver pathology databases, could simplify this process in the future. Utilizing existing linkages between CPRD and the UK Hospital Episodes Statistics database and Office of National Statistics mortality data could have allowed biopsy, scan and mortality data to be considered, which if combined with laboratory results can be used to support the diagnosis of DILI.^10^ In a previous study, however, we found that an algorithm for detecting liver injury that included information on death and 11 different biopsy/scan procedure terms from these data sources provided only very limited improvement on the ability to detect cases (when compared with the use of diagnostic and biochemical criteria from CPRD alone).^22^ The use of our very broad definition (i.e. just jaundice) means that a small degree misclassification of outcome is possible. We used a very thorough process of review to rule out other causes of injury, however, and considered jaundice to be a clear marker of a serious liver problem. Furthermore, the choice not to use the linked datasets meant we had a larger sample size within which our stratified analyses had better power.

Our causal analysis could have been impacted by confounding by indication. To assess the potential for this to occur, we tabulated the 10 most common diagnostic terms entered on the index date for each drug (Supplementary data section 7). For both drugs, the predominant diagnosis was a skin condition—acne for oxytetracycline, cellulitis/skin and subcutaneous tissue infections for flucloxacillin. As cirrhosis is a recognized risk factor for cellulitis,^31^ it is plausible that some of the liver injury observed in flucloxacillin users could be attributed to underlying cirrhosis. We consider this highly unlikely, however, due to the fact that (i) cirrhosis was included as an exclusion term in our study, and (ii) we performed a detailed (blinded) clinician review of medical records in the 6 months prior to the index date to rule out non-drug causes of injury. We also believe that the strength of the association we observe is too large to be explained by confounding by indication. Finally, although we aimed to include participants based upon first-time use of the drugs under study, patients may have been prescribed the drugs prior to registration with a general practice contributing to CPRD, which could mean that our risk estimates are an underestimation of the true frequency within those prescribed flucloxacillin for the first time.

### Conclusions

In the largest known study of flucloxacillin-induced liver injury to date, we have provided new absolute risk estimates by age, number of prescriptions and gender for both laboratory-confirmed injury and jaundice, providing insight into groups particularly susceptible to harm, especially those aged >70 years receiving multiple prescriptions. These results should help guide clinical care decisions and support further work on predictive genetic test implementation.

## Supplementary Material

Supplementary Data
