# Supplementary material for: Quantification of the risk of liver injury associated with flucloxacillin: a UK population-based cohort study
Source: J Antimicrob Chemother. 2017 Jul 1;72(9):2636–46. doi: 10.1093/jac/dkx183 (PMC5890755; doi:10.1093/jac/dkx183)
Supplement: Supplementary Data [file supplementary_data_dkx183.docx]

Supplementary data

1. Choice of comparator drug and information related to data source

Choice of comparator drug - oxytetracycline

Our aim in the causal analysis part of this study was to obtain a comparator population that was (1) similar to the exposed population in terms of health and health-seeking characteristics and (2) was not exposed to a potential hepatotoxin at the start of follow-up. The hepatotoxicity of oxytetracycline itself was not a focus of this study, apart from that it was known to have low hepatotoxicity, so people exposed to it would be likely to experience a risk of liver injury events similar to completely unexposed people. This would allow us to estimate a relative risk of liver injury associated with flucloxacillin use that was as close as possible to the relative risk one would estimate if comparing flucloxacillin users to a group of completely unexposed people with similar health and health-seeking profiles.

Data source - CPRD

The UK Clinical Practice Research Datalink (CPRD Gold, hereafter referred to as CPRD), contains anonymised data on patients from over 625 NHS primary care practices from across the UK (approximately 12 million total patients). Information is recorded by general practitioners or other health centre staff as part of routine clinical care, and data quality checks at the database headquarters ensure that each practice contributing data maintains “up-to-standard” data.[^1^](#_ENREF_1) In addition to the routinely collected data from primary care consultations, information from some secondary sources that has been provided to primary care clinicians (such as major diagnoses made in hospital) may also be recorded. The database has been collecting data since 1987, and has recently been shown to be broadly representative of the UK population.[^2^](#_ENREF_2) Epidemiological research has been performed using the database for over 20 years (generating over 1000 publications),[^3^](#_ENREF_3) and the validity of many diagnoses recorded in the database has been shown to be high.[^1^](#_ENREF_1)

Patient records for study cohorts are extracted from the database based upon the presence of standardised diagnostic codes (Read codes) or, if defining a cohort by drug exposure, British National Formulary (BNF) drug product or substance codes. Searchable dictionaries of all diagnostic and drug prescription terms used in the database are provided, with each record including a specific code and the corresponding descriptive term. Based on the diagnostic or prescription codes selected from the dictionaries, electronic health records can be obtained for all patients who have any of the codes of interest during a period of interest. Data are extracted from the database as a number of separate data files, each containing a different type of data, with the information relevant to any particular patient identified via a unique patient identifier (patient id).

It is usual to ensure that study particpiants all have a minimum prescription history (e.g. 6 months as used in this study) prior to their inclusion in the study, in oder to ensure that the date of prescription for the drug(s) of interest (index date) is as accurate as possible (people who have recently registered may have all legacy diagnoses and prescriptions entered into the system on one date, that represents only the date at which the person was first registered into the system).

1. List of exclusion terms (with their Read codes)

This list was prepared in order to select patients who had any of the following in the 6 months prior to their index date for exclusion: any documented liver disease, jaundice, alcoholism, malignant neoplasm of the liver/gallbladder/pancreas, cholelithiasis, viral hepatitis, chronic liver disease, cirrhosis, congestive heart failure, hepatitis following blood transfusion, HIV, rheumatoid arthritis, sarcoidosis, systemic lupus or inflammatory bowel disease. The diagnostic terms in this list were selected based upon a review of 12 studies[^4-15^](#_ENREF_4) identified by a systematic literature review performed for a previous study on liver injury[^16^](#_ENREF_16). Final review of the included exclusion terms was performed by a member of the study team who is a General Practitioner and Professor in Clinical Epidemiology (LS).

| Term | READ code in lowercase (lc) |
| --- | --- |
| [v]contact with and exposure to viral hepatitis | zv01b00 |
| [v]personal history of alcoholism | zv11300 |
| [v]personal history of malignant neoplasm of liver | zv10015 |
| [v]screening for alcoholism | zv79100 |
| [v]screening for rheumatoid arthritis | zv7y100 |
| [v]viral hepatitis carrier | zv02600 |
| [x]acute alcoholic drunkenness | eu10011 |
| [x]alcoholic dementia nos | eu10711 |
| [x]alcoholic hallucinosis | eu10511 |
| [x]alcoholic jealousy | eu10512 |
| [x]alcoholic paranoia | eu10513 |
| [x]alcoholic psychosis nos | eu10514 |
| [x]chronic alcoholic brain syndrome | eu10712 |
| [x]chronic alcoholism | eu10212 |
| [x]dementia in human immunodef virus [hiv] disease | eu02400 |
| [x]hiv disease result/haematological+immunologic abnorms,nec | ayucb00 |
| [x]hiv disease resulting in multiple infections | ayuc300 |
| [x]hiv disease resulting in other non-hodgkin's lymphoma | ayuc600 |
| [x]hiv disease resulting in other specified conditions | ayucc00 |
| [x]hiv disease resulting/other infectious+parasitic diseases | ayuc400 |
| [x]other and unspecified cirrhosis of liver | jyu7100 |
| [x]other cholecystitis | jyu8100 |
| [x]other cholelithiasis | jyu8000 |
| [x]other crohn's disease | jyu4000 |
| [x]other forms of systemic lupus erythematosus | nyu4300 |
| [x]other seropositive rheumatoid arthritis | nyu1100 |
| [x]other specified acute viral hepatitis | ayub000 |
| [x]other specified rheumatoid arthritis | nyu1200 |
| [x]other ulcerative colitis | jyu4100 |
| [x]rheumatoid arthritis+involvement/other organs or systems | nyu1000 |
| [x]sarcoidosis of other and combined sites | cyu0600 |
| [x]sequelae of viral hepatitis | ayuj900 |
| [x]seropositive rheumatoid arthritis, unspecified | nyu1g00 |
| [x]unspecified human immunodeficiency virus [hiv] disease | ayucd00 |
| [x]viral hepatitis | ayub.00 |
| accidental poisoning by alcoholic beverages | t900.00 |
| acute alcoholic hepatitis | j611.00 |
| acute alcoholic intoxication in alcoholism | e230.00 |
| acute alcoholic intoxication in alcoholism nos | e230z00 |
| acute alcoholic intoxication in remission, in alcoholism | e230300 |
| acute alcoholic intoxication, unspecified, in alcoholism | e230000 |
| acute angiocholecystitis | j650100 |
| acute cholecystitis | j650.00 |
| acute cholecystitis nos | j650z00 |
| acute cholecystitis unspecified | j650000 |
| acute congestive heart failure | g580000 |
| acute emphysematous cholecystitis | j650200 |
| acute gangrenous cholecystitis | j650400 |
| acute polyarticular juvenile rheumatoid arthritis | n043100 |
| acute suppurative cholecystitis | j650300 |
| acute viral hepatitis nos | a70z100 |
| alcohol dependence with acute alcoholic intoxication | e230.11 |
| alcoholic cardiomyopathy | g555.00 |
| alcoholic cirrhosis of liver | j612.00 |
| alcoholic dementia nos | e012.11 |
| alcoholic encephalopathy | f11x011 |
| alcoholic fatty liver | j610.00 |
| alcoholic fibrosis and sclerosis of liver | j612000 |
| alcoholic gastritis | j153.00 |
| alcoholic hepatic failure | j613000 |
| alcoholic hepatitis | j617.00 |
| alcoholic liver damage unspecified | j613.00 |
| alcoholic myopathy | f394100 |
| alcoholic paranoia | e015.00 |
| alcoholic polyneuropathy | f375.00 |
| alcoholic psychoses | e01..00 |
| alcoholic psychosis nos | e01z.00 |
| alcoholics anonymous | 13y8.00 |
| Alcoholism | e23..11 |
| alcoholism counselling | z4b1.00 |
| arthropathy in crohn's disease | n031100 |
| arthropathy in ulcerative colitis | n031000 |
| aversion therapy - alcoholism | 8g32.00 |
| bacterial portal cirrhosis | j615d00 |
| benign neoplasm of gallbladder | b715200 |
| benign neoplasm of liver | b715000 |
| benign neoplasm of liver and biliary ducts | b715.00 |
| bile duct calculus + acute cholecystitis - obstruct nos | j643z00 |
| bile duct calculus + acute cholecystitis and no obstruction | j643000 |
| bile duct calculus + acute cholecystitis and obstruction | j643100 |
| bile duct calculus + other cholecystitis - obstruction nos | j644z00 |
| bile duct calculus + other cholecystitis and obstruction | j644100 |
| bile duct calculus with acute cholecystitis | j643.00 |
| bile duct calculus with other cholecystitis | j644.00 |
| bile duct calculus without cholecystitis nos | j645z00 |
| bile duct calculus without cholecystitis with obstruction | j645100 |
| bile duct calculus without cholecystitis, no obstruction | j645000 |
| bile duct calculus without mention of cholecystitis | j645.00 |
| biliary cirrhosis | j616.00 |
| biliary cirrhosis nos | j616z00 |
| biliary cirrhosis of children | j616200 |
| bmast - brief michigan alcoholism screening test | zra1111 |
| brief michigan alcoholism screening test | zra1100 |
| capsular portal cirrhosis | j615600 |
| carcinoma gallbladder | b160.11 |
| carcinoma in situ of liver | b808000 |
| carcinoma in situ of liver and biliary system | b808.00 |
| carcinoma in situ of liver or biliary system nos | b808z00 |
| carcinoma in situ of pancreas | b80z000 |
| cardiac portal cirrhosis | j615700 |
| cdai - crohn's disease activity index | zr3s.11 |
| cerebellar ataxia due to alcoholism | f144000 |
| cerebral degeneration due to alcoholism | f11x000 |
| cholecystitis nos | j651z00 |
| cholelithiasis | j64..00 |
| cholelithiasis nos | j64z.00 |
| cholelithiasis nos | j64zz00 |
| cholelithiasis with obstruction nos | j64z100 |
| cholelithiasis without obstruction nos | j64z000 |
| chronic alcoholic brain syndrome | e012000 |
| chronic alcoholic hepatitis | j617000 |
| chronic alcoholism | e231.00 |
| chronic alcoholism in remission | e231300 |
| chronic alcoholism nos | e231z00 |
| chronic cholecystitis | j651000 |
| chronic congestive heart failure | g580100 |
| chronic liver disease nos | j61z.00 |
| chronic viral hepatitis | a707.00 |
| chronic viral hepatitis b with delta-agent | a707000 |
| chronic viral hepatitis b without delta-agent | a707100 |
| chronic viral hepatitis c | a707200 |
| chronic viral hepatitis, unspecified | a707x00 |
| cirrhosis - non alcoholic | j615.00 |
| cirrhosis and chronic liver disease | j61..00 |
| cirrhosis of liver nos | j615z13 |
| congenital viral hepatitis | q409.00 |
| congestive heart failure | g580.00 |
| congestive heart failure due to valvular disease | g580400 |
| congestive heart failure monitoring | 662t.00 |
| continuous acute alcoholic intoxication in alcoholism | e230100 |
| continuous chronic alcoholism | e231100 |
| crohn's disease | j40..11 |
| crohn's disease activity index | zr3s.00 |
| crohn's disease nos | j40z.11 |
| crohn's disease of the ileum nos | j400400 |
| crohn's disease of the ileum unspecified | j400300 |
| crohn's disease of the large bowel nos | j401z00 |
| crohn's disease of the small bowel nos | j400z00 |
| crohn's disease of the terminal ileum | j400200 |
| cryptogenic cirrhosis of liver | j615z12 |
| cystic fibrosis related cirrhosis | c370800 |
| cytomegaloviral hepatitis | a785200 |
| delivery of rehabilitation for rheumatoid arthritis | 7p20300 |
| diffuse nodular cirrhosis | j615300 |
| disease activity score 28 joint in rheumatoid arthritis | 38dz000 |
| disease activity score in rheumatoid arthritis | 38dz.00 |
| drug-induced systemic lupus erythematosus | n000200 |
| episodic acute alcoholic intoxication in alcoholism | e230200 |
| episodic chronic alcoholism | e231200 |
| exacerbation of crohn's disease of large intestine | j401200 |
| exacerbation of crohn's disease of small intestine | j400500 |
| exacerbation of ulcerative colitis | j410400 |
| except rheumatoid arthritis qual indicator: informed dissent | 9hr1.00 |
| except rheumatoid arthritis quality indicator: pt unsuitable | 9hr0.00 |
| exception reporting: rheumatoid arthritis quality indicators | 9hr..00 |
| fatty portal cirrhosis | j615400 |
| fh: alcoholism | 1282.00 |
| fh: crohn's disease | 12e5.00 |
| fh: gallbladder disease | 12e4.11 |
| fh: rheumatoid arthritis | 12i1.00 |
| fh: ulcerative colitis | 12e2.11 |
| fibrosing alveolitis associated with rheumatoid arthritis | n04y012 |
| flare of rheumatoid arthritis | n040t00 |
| florid cirrhosis | j612.11 |
| gallbladder calculus with acute cholecystitis | j640.00 |
| gallbladder calculus with acute cholecystitis - obst nos | j640z00 |
| gallbladder calculus with acute cholecystitis + obstruction | j640100 |
| gallbladder calculus with acute cholecystitis +no obstruct | j640000 |
| gallbladder calculus with other cholecystitis | j641.00 |
| gallbladder calculus with other cholecystitis - obstruct nos | j641z00 |
| gallbladder calculus with other cholecystitis + obstruct | j641100 |
| gallbladder calculus with other cholecystitis +no obstruct | j641000 |
| gallbladder calculus without cholecystitis and obstruct nos | j642z00 |
| gallbladder calculus without mention cholecystitis + obstruc | j642100 |
| gallbladder calculus without mention cholecystitis +no obstr | j642000 |
| gallbladder calculus without mention of cholecystitis | j642.11 |
| gallbladder calculus without mention of cholecystitis | j642.00 |
| gallstones | j64..15 |
| glycogenosis with hepatic cirrhosis | c310400 |
| h/o: alcoholism | 1462.00 |
| h/o: gallbladder disease | 14c7.11 |
| h/o: rheumatoid arthritis | 14g1.00 |
| h/o: ulcerative colitis | 14c4.11 |
| hepatic granulomas in sarcoidosis | j63a.00 |
| hepatoblastoma of liver | b150100 |
| history of viral hepatitis | 141f.00 |
| hiv disease complicating pregnancy childbirth puerperium | l179.00 |
| hiv disease result/haematological+immunologic abnorms,nec | a788u00 |
| hiv disease resulting in burkitt's lymphoma | a789600 |
| hiv disease resulting in candidiasis | a789200 |
| hiv disease resulting in cytomegaloviral disease | a789100 |
| hiv disease resulting in kaposi's sarcoma | a789500 |
| hiv disease resulting in lymphoid interstitial pneumonitis | a789900 |
| hiv disease resulting in multiple infections | a789400 |
| hiv disease resulting in multiple malignant neoplasms | a789800 |
| hiv disease resulting in mycobacterial infection | a789000 |
| hiv disease resulting in pneumocystis carinii pneumonia | a789300 |
| hiv disease resulting in pneumocystis jirovecii pneumonia | a789311 |
| hiv disease resulting in unspecified malignant neoplasm | a788w00 |
| hiv disease resulting in wasting syndrome | a789a00 |
| hiv disease resulting/unspcf infectious+parasitic disease | a788x00 |
| hiv infection with persistent generalised lymphadenopathy | a788200 |
| hiv positive | 43c3.11 |
| husband alcoholic | 13l3.13 |
| hypertrophic portal cirrhosis | j615500 |
| indian childhood cirrhosis | j615812 |
| infectious cirrhosis nos | j615h00 |
| inflammatory bowel disease | j4...12 |
| [d]jaundice (not of newborn) | r024.00 |
| [d]jaundice | r024111 |
| obstructive jaundice nos | j66y600 |
| o/e – jaundiced | 2274.11 |
| jaundice – symptom | 1675.11 |
| [d]icterus nos | r024100 |
| yellow/jaundiced colour | 1675.00 |
| o/e - jaundiced colour | 2274.00 |
| [d]jaundice (not of newborn) nos | r024z00 |
| juvenile arthritis in crohn's disease | n045300 |
| juvenile arthritis in ulcerative colitis | n045400 |
| juvenile portal cirrhosis | j615800 |
| juvenile rheumatoid arthritis | n045500 |
| juvenile rheumatoid arthritis - still's disease | n043.00 |
| juvenile rheumatoid arthritis nos | n043z00 |
| korsakoff's non-alcoholic psychosis | e040.11 |
| korsakov's alcoholic psychosis | e011000 |
| korsakov's alcoholic psychosis with peripheral neuritis | e011100 |
| laennec's cirrhosis | j612.12 |
| liver abscess and sequelae of chronic liver disease | j62..00 |
| liver metastases | b577.11 |
| lung disease with systemic lupus erythematosus | h57y400 |
| macronodular cirrhosis of liver | j615z11 |
| malignant neoplasm gallbladder and extrahepatic bile ducts | b16..00 |
| malignant neoplasm gallbladder/extrahepatic bile ducts nos | b16z.00 |
| malignant neoplasm of body of pancreas | b171.00 |
| malignant neoplasm of gallbladder | b160.00 |
| malignant neoplasm of head of pancreas | b170.00 |
| malignant neoplasm of liver and intrahepatic bile ducts | b15..00 |
| malignant neoplasm of liver and intrahepatic bile ducts nos | b15z.00 |
| malignant neoplasm of liver unspecified | b152.00 |
| malignant neoplasm of other specified sites of pancreas | b17y.00 |
| malignant neoplasm of pancreas | b17..00 |
| malignant neoplasm of pancreas nos | b17z.00 |
| malignant neoplasm of specified site of pancreas nos | b17yz00 |
| malignant neoplasm of tail of pancreas | b172.00 |
| malignant neoplasm other gallbladder/extrahepatic bile duct | b16y.00 |
| mast - michigan alcoholism screening test | zra1.11 |
| meningitis due to sarcoidosis | f013.00 |
| michigan alcoholism screening test | zra1.00 |
| monarticular juvenile rheumatoid arthritis | n043300 |
| multilobular portal cirrhosis | j615100 |
| multiple cranial nerve palsies in sarcoidosis | f326300 |
| munich alcoholism test | zrau.00 |
| myopathy due to rheumatoid arthritis | f396400 |
| myopathy due to sarcoidosis | f396500 |
| myositis in sarcoidosis | n233200 |
| neoplasm of uncertain behaviour of liver | b903000 |
| neoplasm of uncertain behaviour of liver and biliary passage | b903.00 |
| nephrotic syndrome in systemic lupus erythematosus | k01x400 |
| non-alcoholic cirrhosis nos | j615z00 |
| non-alcoholic fatty liver | j61y100 |
| o/e - alcoholic breath | 2577.11 |
| oesophageal varices in alcoholic cirrhosis of the liver | g852300 |
| oesophageal varices in cirrhosis of the liver | g852200 |
| orofacial crohn's disease | j08z900 |
| other alcoholic dementia | e012.00 |
| other alcoholic psychosis | e01y.00 |
| other alcoholic psychosis nos | e01yz00 |
| other cholecystitis | j651.00 |
| other cholecystitis os | j651y00 |
| other non-alcoholic chronic liver disease | j61y.00 |
| other non-alcoholic chronic liver disease nos | j61yz00 |
| other rheumatoid arthritis of spine | n040100 |
| other sequelae of chronic liver disease | j62y.00 |
| other specified viral hepatitis with coma | a704.00 |
| other specified viral hepatitis with hepatic coma nos | a704z00 |
| other specified viral hepatitis without coma | a705.00 |
| other specified viral hepatitis without mention of coma nos | a705z00 |
| pauciarticular juvenile rheumatoid arthritis | n043200 |
| pigmentary cirrhosis of liver | c350012 |
| polyneuropathy in rheumatoid arthritis | f371200 |
| polyneuropathy in sarcoidosis | f374900 |
| portal cirrhosis | j615.11 |
| portal cirrhosis unspecified | j615y00 |
| portal fibrosis without cirrhosis | j61y300 |
| primary biliary cirrhosis | j616000 |
| primary carcinoma of liver | b150000 |
| primary malignant neoplasm of liver | b150.00 |
| primary malignant neoplasm of liver nos | b150z00 |
| pulmonary sarcoidosis | h57y200 |
| regional enteritis - crohn's disease | j40..00 |
| rheumatoid arthritis | n040.00 |
| rheumatoid arthritis - multiple joint | n040s00 |
| rheumatoid arthritis and other inflammatory polyarthropathy | n04..00 |
| rheumatoid arthritis annual review | 66hb000 |
| rheumatoid arthritis of 1st mtp joint | n040k00 |
| rheumatoid arthritis of acromioclavicular joint | n040400 |
| rheumatoid arthritis of ankle | n040f00 |
| rheumatoid arthritis of cervical spine | n040000 |
| rheumatoid arthritis of dip joint of finger | n040a00 |
| rheumatoid arthritis of distal radio-ulnar joint | n040600 |
| rheumatoid arthritis of elbow | n040500 |
| rheumatoid arthritis of hip | n040b00 |
| rheumatoid arthritis of knee | n040d00 |
| rheumatoid arthritis of lesser mtp joint | n040l00 |
| rheumatoid arthritis of mcp joint | n040800 |
| rheumatoid arthritis of other tarsal joint | n040j00 |
| rheumatoid arthritis of pip joint of finger | n040900 |
| rheumatoid arthritis of sacro-iliac joint | n040c00 |
| rheumatoid arthritis of shoulder | n040200 |
| rheumatoid arthritis of subtalar joint | n040g00 |
| rheumatoid arthritis of talonavicular joint | n040h00 |
| rheumatoid arthritis of wrist | n040700 |
| rheumatoid arthritis particle agglutination test | 43b9.00 |
| rheumatoid arthritis screen | 68f1.00 |
| rheumatoid arthritis screening test | 43c6.00 |
| sarcoidosis | ad5..00 |
| sarcoidosis of inferior turbinates | ad54.00 |
| sarcoidosis of lung | ad50.00 |
| sarcoidosis of lung with sarcoidosis of lymph nodes | ad52.00 |
| sarcoidosis of lymph nodes | ad51.00 |
| sarcoidosis of skin | ad53.00 |
| secondary biliary cirrhosis | j616100 |
| secondary malignant neoplasm of liver | b577.00 |
| secondary malignant neoplasm of liver | b153.00 |
| sequelae of viral hepatitis | ae23.00 |
| seronegative rheumatoid arthritis | n040p00 |
| seropositive errosive rheumatoid arthritis | n047.00 |
| seropositive rheumatoid arthritis, unspecified | n04x.00 |
| slam - systemic lupus activity measure | zrq8.11 |
| suspected gallstones | 1j5..00 |
| systemic lupus activity measure | zrq8.00 |
| systemic lupus erythematosus | n000.00 |
| systemic lupus erythematosus disease activity index | zrq9.00 |
| systemic lupus erythematosus nos | n000z00 |
| systemic lupus erythematosus with organ or sys involv | n000300 |
| systemic lupus erythematosus with pericarditis | n000400 |
| toxic liver disease with fibrosis and cirrhosis of liver | j635600 |
| ulcerative colitis | j410100 |
| ulcerative colitis and/or proctitis | j41..12 |
| unspecified chronic alcoholism | e231000 |
| unspecified viral hepatitis | a70z.00 |
| viral (serum) hepatitis b | a703.00 |
| viral hepatitis | a70..00 |
| viral hepatitis a with coma | a700.00 |
| viral hepatitis b with coma | a702.00 |
| viral hepatitis c with coma | a704000 |
| viral hepatitis c without mention of hepatic coma | a705000 |
| viral hepatitis carrier | 65q7.00 |
| viral hepatitis comp pregnancy, childbirth & the puerperium | l176500 |
| viral hepatitis screening test | 4jrf.00 |
| viral hepatitis without hepatic coma | a709.00 |
| xanthomatous portal cirrhosis | j615c00 |

1. Case definition

3a Diagnostic terms indicating liver injury

(a) Search terms used to search for relevant diagnostic terms in CPRD diagnosis dictionary

| **Inclusion terms** |
| --- |
| **Search based on the word “liver”**  *liver* AND (*biopsy* OR *necrosis* OR *disease* OR * enlarged* OR *disorder*) |
| **Search based on the word “hepatic”**  *hepatic* AND (*failure* OR *coma* OR encephalopathy*) |
| **Other search terms**  *cholesta*, *jaundice*, *icterus*, *cholangitis*, *other gall bladder disorders*, *cholaemia*, *yellow atrophy*, *hepatitis* |
| **Terms excluded during search to increase specificity** |
| *fetal*, *hepatitis a*, *hepatitis b*, *hepatitis c*, *hepatitis e*, *hepatitis g*, *delive*, *pregn*, *neonat*, *perinatal*, *viral*, *virus*, *congenital*, *autoimmune* |

**Note 1:** * represents a wildcard, which means that any text can be present in this position

**Note 2:** the search was set to look for words after the word “AND” on either side of the main search term (e.g. both “liver biopsy” and “biopsy liver” would be searched for)

(b) CPRD terms selected following search

| Term | READ code (lc) |
| --- | --- |
| toxic liver disease with cholestasis | j635000 |
| [d]jaundice (not of newborn)* | r024.00 |
| hepatitis unspecified | j633.00 |
| [d]jaundice* | r024111 |
| obstructive jaundice nos* | j66y600 |
| hepatitis unspecified nos | j633z00 |
| o/e – jaundiced* | 2274.11 |
| jaundice – symptom* | 1675.11 |
| [d]icterus nos* | r024100 |
| yellow/jaundiced colour* | 1675.00 |
| o/e - jaundiced colour* | 2274.00 |
| [d]jaundice (not of newborn) nos* | r024z00 |
| infective hepatitis | a701.11 |
| other liver disorders | j63..00 |
| chronic hepatitis | j614.00 |
| chronic aggressive hepatitis | j614200 |
| acute alcoholic hepatitis | j611.00 |
| other specified liver disorder nos | j63yz00 |
| [d]cholaemia nos | r024000 |
| acute hepatic failure | j600000 |
| o/e - liver grossly enlarged | 25g4.00 |
| alcoholic hepatitis | j617.00 |
| open wedge biopsy of lesion of liver | 7804200 |
| Cholangitis | j661.00 |
| biopsy of liver nec | 780b000 |
| liver disorder nos | j63z.00 |
| primary sclerosing cholangitis | j661700 |
| chronic hepatitis nos | j614z00 |
| toxic hepatitis | j633000 |
| recurrent cholangitis | j661200 |
| acute hepatitis – noninfective | j600100 |
| o/e -liver moderately enlarged | 25g3.00 |
| hepatic failure | j62y.13 |
| needle biopsy of liver nec | 780a112 |
| toxic liver disease with chronic persistent hepatitis | j635300 |
| alcoholic hepatic failure | j613000 |
| hepatitis non a non b | a705400 |
| fh: hepatitis | 12e3.11 |
| subacute hepatitis – noninfective | j601100 |
| encephalopathy – hepatic | j622.11 |
| hepatic coma | j622.00 |
| chronic persistent hepatitis | j614000 |
| calculus of bile duct with cholangitis | j646.00 |
| [x] hepatic failure | j625.00 |
| liver abscess due to cholangitis | j620100 |
| other specified liver disorder | j63y.00 |
| subacute hepatic failure | j601000 |
| nonspecific reactive hepatitis | j63y100 |
| other cholangitis | j661y00 |
| other non-alcoholic chronic liver disease nos | j61yz00 |
| ascending cholangitis | j661400 |
| percutaneous transvascular biopsy of lesion of liver | 780a000 |
| toxic liver disease with hepatic necrosis | j635100 |
| hepatic failure as a complication of care | sp14200 |
| toxic liver disease | j635.00 |
| toxic liver disease with chronic active hepatitis | j635500 |
| toxic liver disease with acute hepatitis | j635200 |
| acute hepatic failure due to drugs | j635700 |
| acute necrosis of liver | j600.00 |
| toxic liver disease, unspecified | j635x00 |
| other non-alcoholic chronic liver disease | j61y.00 |
| toxic liver disease with fibrosis and cirrhosis of liver | j635600 |
| sclerosing cholangitis unspecified | j661900 |
| other sequelae of chronic liver disease | j62y.00 |
| acute and subacute liver necrosis | j60..00 |
| chronic cholangitis | j661100 |
| recurrent hepatitis | j614300 |
| acute yellow atrophy | j600200 |
| chronic hepatitis unspecified | j614y00 |
| acute necrosis of liver nos | j600z00 |
| hepatic failure nos | j62y.11 |
| subacute necrosis of liver | j601.00 |
| cholangitis nos | j661z00 |
| central haemorrhagic necrosis of liver | j636.00 |
| toxic liver disease with chronic lobular hepatitis | j635400 |
| menghini needle biopsy of liver | 780a111 |
| acute and subacute liver necrosis nos | j60z.00 |
| toxoplasma hepatitis | ad05.00 |
| cholangitis lenta | j661500 |
| chronic lobular hepatitis | j614400 |
| subacute yellow atrophy | j601200 |
| subacute necrosis of liver nos | j601z00 |
| endoscopic ultrasound examination liver biopsy lesion liver | 780f000 |
| obliterative cholangitis | j661600 |
| sheeba needle biopsy of liver | 780a113 |

*****Terms/codes required for an individual to be a symptom-defined case. For laboratory-confirmed cases, the individual could have any of the codes in this list (provided they also had a laboratory test results indicative of DILI).

3b Comparison of selected cases with the RUCAM/CIOMS causality assessment method

For each of the cases we collected information on the type of liver injury, whether exposure was first or second, time from drug intake until reaction onset, presence of alcohol or pregnancy as a risk factor, age >=55 as a risk factor, presence and nature of concomitant therapy, whether non drug-related causes had been ruled out and prescribing information related to flucloxacillin hepatotoxicity in order to test each case against the RUCAM/CIOMS criteria for assessing causality of drug-induced liver injury.[^17^](#_ENREF_17) Additional information that this method can use relates to course of the reaction (i.e. how the patient improved over the following 180 days) and response to re-administration, data that was not available within CPRD. The RUCAM/CIOMS causality method uses all of this information to derive a score, which is converted into the following categories:

- ≤0: Excluded ADR*
- 1-2: Unlikely ADR
- 3-5: Possible ADR
- 6-8: Probable ADR
- ≥9: Highly probable ADR

*ADR caused by drug of interest

Given that information on course of the reaction or response to re-administration was not available to us, the highest score that any of our cases could attain was an 8 (Probable ADR).

1. Selection and management of covariates, and handling of missing data
2. **Selection and management of covariates**

A causal diagram was used in order to inform the selection of covariates.[^18^](#_ENREF_18) This was prepared using the DAGittty graphical tool for analyzing causal diagrams,[^19^](#_ENREF_19) and is reproduced below.


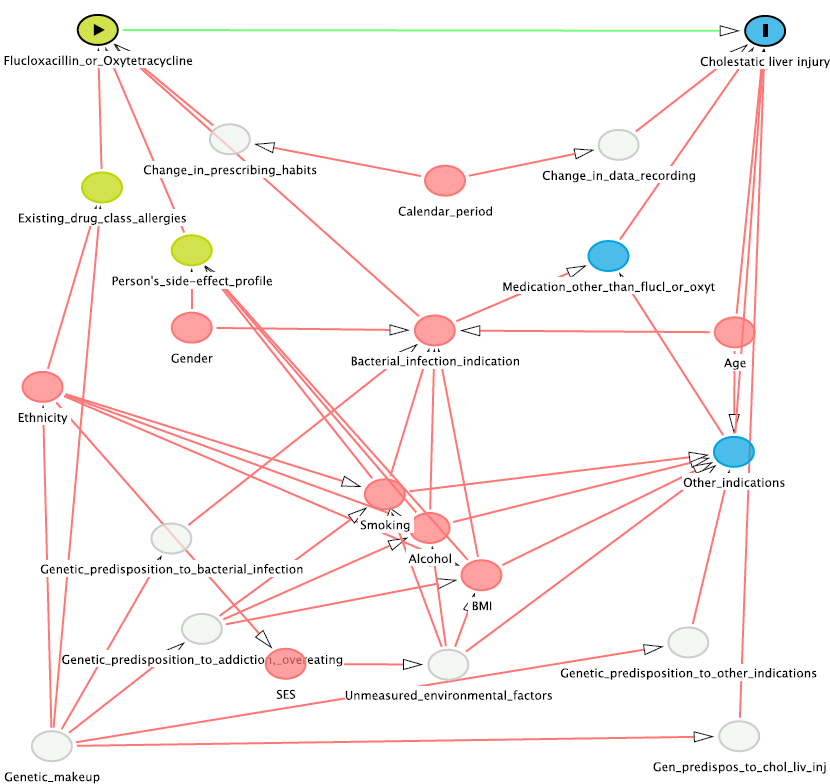


Age and gender have been suggested as risk factors for flucloxacillin-induced liver injury,[^14^](#_ENREF_14)^,^ [^20^](#_ENREF_20)^,^ [^21^](#_ENREF_21) and are included as part of the CPRD patient (demographic) data file. A categorical age variable was created with ten year categories.

The role of ethnicity has not been previously studied, and was considered important due to a likely genetic susceptibility to flucloxacillin-induced liver injury.[^22^](#_ENREF_22)^,^ [^23^](#_ENREF_23) Ethnicity codes based on the 2001 UK census data were used to search the CPRD additional files,[^24^](#_ENREF_24) in order to assign ethnicity to the cohort, as a 5-category variable. If no ethnicity records were found in CPRD for a patient, the HES records of any patients that were HES-linked were also searched for ethnicity information. Any patients who did not have ethnicity information in CPRD or HES were assigned to an “Unknown” category.

BMI, alcohol intake and SES are likely to be associated with indications requiring flucloxacillin (such as cellulitis) and with susceptibility to liver-related conditions. CPRD smoking status is recorded as non-, ex-, current and unknown. CPRD alcohol status is recorded as never, ex-, current (not-otherwise-specified), ≤2 or less units/day, 3-6 units/day, >6 units per day, and unknown. A categorical BMI status variable was created with categories of <20, 20-25, 25+, and unknown. Smoking, alcohol and BMI status were assigned according to the classification of the nearest date prior to the index date (if no prior status, the status from the nearest post-index date was used). SES information is not part of the standard CPRD database, and was obtained separately as linked data. This was provided by CPRD as an Index of Multiple Deprivation score based upon individual patient postcode, and as a practice level score based upon practice postcode (both variables consisting of 5 categories representing quintiles of score). A dedicated SES variable was created for the study and populated with the patient-level score, unless this was missing, in which case the practice-level score was used. This linked SES data was only available for practices in England.

Calendar period was included in order to assess if changes in prescribing habits or recording of outcomes occurred over time (for example, improved automation of the system for capturing liver test results within CPRD over time). A calendar time-period variable was created with categories spanning 3-year periods.

The possible impact of the use of other drugs associated with liver injury was assessed by looking for prescriptions for other drugs that occurred before the end of follow-up for the patient and up to 1 month before the index date. A variable was created with three categories: 0=no use of other drugs, 1=use of drugs thought to cause liver injury at a frequency lower than flucloxacillin, 2=use of drugs thought to cause liver injury at a frequency higher than flucloxacillin. The list of drugs and categorisation was based upon a systematic literature review of epidemiological studies of drug-induced liver injury performed for a previous study on liver injury,[^16^](#_ENREF_16) combined with information from three reviews relating to drug-induced liver injury[^25-27^](#_ENREF_25) and from the LIVERTOX website.[^28^](#_ENREF_28) The drugs included NSAIDs, other antibiotics, antidepressants and antifungals, and a full list of therapies is provided below.

| Drug substance | Frequency category(category=less frequent unless marked) |
| --- | --- |
| acebutolol hydrochloride/hydrochlorothiazide |  |
| allopurinol |  |
| aluminium hydroxide/bismuth subnitrate/magnesium carbonate/sodium bicarbonate/deglycyrrhizinised liquorice |  |
| amiloride hydrochloride/hydrochlorothiazide |  |
| amiodarone hydrochloride |  |
| amitriptyline hydrochloride |  |
| amitriptyline hydrochloride/perphenazine |  |
| amlodipine |  |
| amlodipine besilate |  |
| amlodipine besilate/hydrochlorothiazide/olmesartan medoxomil |  |
| amlodipine besilate/olmesartan medoxomil |  |
| amlodipine besilate/valsartan |  |
| amoxicillin sodium |  |
| amoxicillin sodium/potassium clavulanate | More frequent |
| amoxicillin trihydrate |  |
| amoxicillin trihydrate/potassium clavulanate | More frequent |
| ampicillin |  |
| ampicillin sodium |  |
| ampicillin trihydrate |  |
| aspirin |  |
| aspirin/aloxiprin/caffeine citrate |  |
| aspirin/caffeine |  |
| aspirin/caffeine/citric acid/sodium bicarbonate/paracetamol |  |
| aspirin/codeine phosphate |  |
| aspirin/ethoheptazine citrate/meprobamate |  |
| aspirin/isosorbide mononitrate |  |
| aspirin/papaveretum |  |
| aspirin/paracetamol |  |
| aspirin/paracetamol/caffeine |  |
| atenolol/amiloride hydrochloride/hydrochlorothiazide |  |
| atorvastatin calcium |  |
| atorvastatin calcium trihydrate |  |
| auranofin |  |
| azathioprine | More frequent |
| bacampicillin |  |
| benzoyl peroxide/clindamycin phosphate |  |
| benzoyl peroxide/erythromycin |  |
| betahistine dihydrochloride |  |
| bezafibrate |  |
| bisoprolol fumarate/hydrochlorothiazide |  |
| bupropion hydrochloride |  |
| captopril |  |
| carbamazepine | More frequent |
| carmustine |  |
| cefalexin |  |
| cefuroxime |  |
| cefuroxime axetil |  |
| cefuroxime sodium |  |
| celecoxib |  |
| cetirizine hydrochloride |  |
| chlorambucil |  |
| chlorothiazide |  |
| chlorpromazine embonate | More frequent |
| chlorpromazine hydrochloride | More frequent |
| chlortetracycline hydrochloride |  |
| chlortetracycline hydrochloride/tetracycline hydrochloride/demeclocycline hydrochloride |  |
| chlortetracycline hydrochloride/triamcinolone acetonide |  |
| cimetidine |  |
| cinnarizine |  |
| ciprofloxacin |  |
| ciprofloxacin hydrochloride |  |
| ciprofloxacin lactate |  |
| clarithromycin |  |
| clindamycin hydrochloride |  |
| clindamycin phosphate |  |
| clobetasone butyrate/oxytetracycline calcium/nystatin |  |
| clomethiazole |  |
| clomethiazole edisilate |  |
| clomipramine hydrochloride |  |
| clopidogrel |  |
| clopidogrel hydrogen sulphate |  |
| cloxacillin |  |
| clozapine |  |
| codeine phosphate/aspirin |  |
| cyclophosphamide |  |
| cyclophosphamide monohydrate |  |
| cyproheptadine hydrochloride |  |
| danazol |  |
| desogestrel/ethinylestradiol |  |
| dexibuprofen |  |
| dexketoprofen trometamol |  |
| dextromethorphan hydrobromide |  |
| dextromethorphan hydrobromide/ephedrine hydrochloride |  |
| dextromethorphan hydrobromide/menthol |  |
| dextromethorphan hydrobromide/pseudoephedrine hydrochloride |  |
| dextromethorphan hydrobromide/terpin hydrate/menthol/pumilio pine oil/eucalyptus oil |  |
| dextromethorphan hydrobromide/triprolidine hydrochloride |  |
| dextropropoxyphene hydrochloride |  |
| dextropropoxyphene hydrochloride/paracetamol |  |
| dextropropoxyphene napsylate |  |
| diazepam |  |
| diclofenac diethylammonium |  |
| diclofenac epolamine |  |
| diclofenac potassium |  |
| diclofenac sodium |  |
| diclofenac sodium/misoprostol |  |
| diflunisal |  |
| digoxin |  |
| dimenhydrinate/cinnarizine |  |
| diphenhydramine hydrochloride/dextromethorphan hydrobromide |  |
| diphenhydramine hydrochloride/menthol/dextromethorphan hydrobromide |  |
| dipyridamole/aspirin |  |
| disulfiram |  |
| dosulepin hydrochloride |  |
| doxazosin mesilate |  |
| doxycycline hyclate |  |
| doxycycline monohydrate |  |
| drospirenone/estradiol hemihydrate |  |
| drospirenone/ethinylestradiol |  |
| duloxetine hydrochloride |  |
| dydrogesterone/estradiol |  |
| enalapril maleate |  |
| enalapril maleate/hydrochlorothiazide |  |
| erythromycin |  |
| erythromycin ethyl succinate |  |
| erythromycin lactobionate |  |
| erythromycin stearate |  |
| erythromycin/isotretinoin |  |
| erythromycin/tretinoin |  |
| erythromycin/zinc acetate |  |
| escitalopram oxalate |  |
| esomeprazole magnesium |  |
| esomeprazole magnesium dihydrate |  |
| esomeprazole magnesium trihydrate |  |
| esomeprazole sodium |  |
| estradiol |  |
| estradiol acetate |  |
| estradiol hemihydrate |  |
| estradiol valerate |  |
| estradiol valerate/norethisterone |  |
| estradiol/levonorgestrel |  |
| estradiol/norethisterone acetate |  |
| estrone/estriol/estradiol |  |
| ethinylestradiol |  |
| ethinylestradiol/cyproterone acetate |  |
| ethinylestradiol/etonogestrel |  |
| etodolac |  |
| etoricoxib |  |
| ezetimibe/simvastatin |  |
| fenofibrate |  |
| fenofibrate micronised |  |
| fenoprofen calcium |  |
| flucloxacillin magnesium |  |
| flucloxacillin magnesium/ampicillin trihydrate |  |
| flucloxacillin sodium |  |
| flucloxacillin sodium/ampicillin trihydrate |  |
| fluconazole |  |
| fluphenazine decanoate |  |
| fluphenazine enantate |  |
| fluphenazine hydrochloride |  |
| fluphenazine hydrochloride/nortriptyline hydrochloride |  |
| flutamide |  |
| fluvastatin sodium |  |
| fosinopril sodium |  |
| fosphenytoin sodium |  |
| gabapentin |  |
| gemcitabine hydrochloride |  |
| gestodene/ethinylestradiol |  |
| glibenclamide |  |
| glimepiride |  |
| griseofulvin |  |
| haloperidol |  |
| haloperidol decanoate |  |
| halothane |  |
| hydrochlorothiazide |  |
| hydrochlorothiazide/amlodipine besilate/olmesartan medoxomil |  |
| hydrochlorothiazide/amlodipine/olmesartan medoxomil |  |
| hydrochlorothiazide/captopril |  |
| hydrochlorothiazide/irbesartan |  |
| hydrochlorothiazide/losartan potassium |  |
| hydrochlorothiazide/metoprolol tartrate |  |
| hydrochlorothiazide/olmesartan medoxomil |  |
| hydrochlorothiazide/quinapril hydrochloride |  |
| hydrochlorothiazide/telmisartan |  |
| hydrochlorothiazide/valsartan |  |
| hydrocortisone/nystatin/oxytetracycline calcium |  |
| ibuprofen |  |
| ibuprofen lysine |  |
| ibuprofen sodium dihydrate |  |
| ibuprofen/codeine phosphate |  |
| ibuprofen/levomenthol |  |
| ibuprofen/paracetamol |  |
| ibuprofen/phenylephrine hydrochloride |  |
| ibuprofen/pseudoephedrine hydrochloride |  |
| imipramine hydrochloride |  |
| infliximab |  |
| interferon beta-1a |  |
| iproniazide |  |
| irbesartan |  |
| irbesartan/hydrochlorothiazide |  |
| isoflurane |  |
| isoniazid | More frequent |
| itraconazole |  |
| ketoprofen |  |
| ketoprofen/omeprazole |  |
| ketorolac trometamol |  |
| lamotrigine |  |
| leflunomide |  |
| levocetirizine dihydrochloride |  |
| levofloxacin |  |
| levofloxacin hemihydrate |  |
| levonorgestrel |  |
| levonorgestrel/ethinylestradiol |  |
| linagliptin/metformin hydrochloride |  |
| lisinopril |  |
| lisinopril/hydrochlorothiazide |  |
| lithium carbonate |  |
| lithium citrate |  |
| losartan potassium/hydrochlorothiazide |  |
| loxapine succinate |  |
| lysine acetylsalicylate/metoclopramide hydrochloride | More frequent |
| medroxyprogesterone acetate/estradiol valerate |  |
| mefenamic acid |  |
| meloxicam |  |
| mepivacaine hydrochloride/nicotinamide/polyestradiol phosphate |  |
| mercaptopurine |  |
| metformin |  |
| metformin hydrochloride |  |
| metformin hydrochloride/rosiglitazone maleate |  |
| metformin hydrochloride/saxagliptin hydrochloride |  |
| metformin hydrochloride/sitagliptin phosphate |  |
| metformin hydrochloride/vildagliptin |  |
| methyldopa anhydrous |  |
| methyldopate hydrochloride |  |
| methyltestosterone |  |
| methyltestosterone/pemoline/yohimbine hydrochloride |  |
| metoclopramide hydrochloride | More frequent |
| metoclopramide hydrochloride/paracetamol | More frequent |
| metoprolol tartrate/hydrochlorothiazide |  |
| mianserin hydrochloride |  |
| minocycline hydrochloride |  |
| nabumetone |  |
| nandrolone decanoate |  |
| naproxen |  |
| naproxen sodium |  |
| naproxen/esomeprazole |  |
| nefazodone hydrochloride |  |
| nevirapine |  |
| nevirapine anhydrate |  |
| nevirapine hemihydrate |  |
| nitrofurantoin |  |
| nomegestrol/estradiol hemihydrate |  |
| norelgestromin/ethinylestradiol |  |
| norethisterone acetate/estradiol |  |
| norethisterone acetate/ethinylestradiol |  |
| norethisterone/ethinylestradiol |  |
| norfloxacin |  |
| norgestimate/ethinylestradiol |  |
| olanzapine |  |
| olanzapine embonate monohydrate |  |
| olmesartan medoxomil/amlodipine besilate |  |
| olmesartan medoxomil/hydrochlorothiazide |  |
| omeprazole |  |
| omeprazole magnesium |  |
| omeprazole sodium |  |
| orlistat |  |
| oxandrolone |  |
| oxymetholone |  |
| oxytetracycline dihydrate |  |
| oxytetracycline hydrochloride |  |
| oxytetracycline hydrochloride/hydrocortisone |  |
| paracetamol/caffeine/aspirin |  |
| paracetamol/dextropropoxyphene hydrochloride |  |
| paracetamol/ibuprofen |  |
| paracetamol/metoclopramide hydrochloride | More frequent |
| paracetamol/promethazine hydrochloride/dextromethorphan hydrobromide |  |
| paracetamol/pseudoephedrine hydrochloride/doxylamine succinate/dextromethorphan hydrobromide |  |
| paroxetine hydrochloride |  |
| perphenazine |  |
| phenoxymethylpenicillin potassium |  |
| phenylbutazone |  |
| phenytoin |  |
| phenytoin sodium |  |
| pimozide |  |
| pioglitazone hydrochloride |  |
| pioglitazone hydrochloride/metformin hydrochloride |  |
| piroxicam |  |
| piroxicam betadex |  |
| pivampicillin |  |
| polymyxin b sulphate/trimethoprim |  |
| prochlorperazine maleate |  |
| prochlorperazine mesilate |  |
| propafenone hydrochloride |  |
| pseudoephedrine hydrochloride/dextromethorphan hydrobromide |  |
| pseudoephedrine hydrochloride/ibuprofen |  |
| pseudoephedrine hydrochloride/levomenthol/diphenhydramine hydrochloride/dextromethorphan hydrobromide |  |
| pseudoephedrine hydrochloride/triprolidine hydrochloride/dextromethorphan hydrobromide |  |
| pyrazinamide/rifampicin/isoniazid | More frequent |
| quetiapine fumarate |  |
| racemic camphor/aspirin/methyl salicylate/menthol |  |
| ranitidine bismuth citrate |  |
| ranitidine hydrochloride |  |
| repaglinide |  |
| rifampicin |  |
| rifampicin/isoniazid | More frequent |
| risperidone |  |
| rofecoxib |  |
| rosiglitazone maleate |  |
| rosiglitazone maleate/metformin hydrochloride |  |
| sertraline hydrochloride |  |
| simvastatin |  |
| simvastatin/ezetimibe |  |
| sodium aurothiomalate |  |
| sodium fusidate |  |
| sodium fusidate/hydrocortisone acetate |  |
| sodium valproate | More frequent |
| spironolactone/chlorothiazide |  |
| stanozolol |  |
| sulfamethoxazole/trimethoprim |  |
| sulindac |  |
| sulpiride | More frequent |
| talampicillin hydrochloride |  |
| tamoxifen citrate |  |
| telmisartan/hydrochlorothiazide |  |
| tenoxicam |  |
| terbinafine |  |
| terbinafine hydrochloride |  |
| terfenadine |  |
| testosterone |  |
| testosterone enantate |  |
| testosterone phenylpropionate/testosterone propionate/testosterone decanoate/testosterone isocaproate |  |
| testosterone propionate |  |
| testosterone propionate/testosterone phenylpropionate/testosterone isocaproate |  |
| testosterone undecanoate |  |
| tetracycline hydrochloride |  |
| thioridazine |  |
| thioridazine hydrochloride |  |
| ticlopidine hydrochloride |  |
| timolol maleate/hydrochlorothiazide/amiloride hydrochloride |  |
| tolbutamide |  |
| tolmetin sodium |  |
| triamterene/hydrochlorothiazide |  |
| trifluoperazine hydrochloride |  |
| trimethoprim |  |
| trimethoprim/sulfamethoxazole |  |
| trimipramine maleate |  |
| valproate semisodium | More frequent |
| valsartan/amlodipine besilate |  |
| valsartan/hydrochlorothiazide |  |
| vildagliptin/metformin hydrochloride |  |
| zinc sulphate/lithium succinate |  |

1. **Handling of missing data**

In order to allow multivariable analysis of the association between flucloxacillin and liver injury (compared with oxytetracycline), missing data were accounted for using multiple imputation by chained equations, assuming a missing at random (MAR) model of missingness.[^29^](#_ENREF_29) All the variables included in Table 2 were included in the imputation model, in addition to outcome status. Five imputed datasets were created and combined for analysis, and the results of complete records analysis and multiple imputed analysis were tabulated as part of the results.

1. Supplementary Data Results Tables

Table S1 - For the 1-90 period from first prescription: (1) the number of cases of liver injury within people prescribed flucloxacillin or oxytetracycline (2) the median time from first recorded flucloxacillin prescription until case assignment and (3) characteristics of symptom-defined and laboratory-confirmed liver injury cases in the exposed to flucloxacillin group

|  |  | Flucloxacillin or oxytetracycline |  | Flucloxacillin-only | |  | |
| --- | --- | --- | --- | --- | --- | --- | --- |
| **Case definition^1^** |  | **Number of (potential) liver injury cases: number subsequently identified as flucl- or oxyt- induced (%)^2^** |  | **Time from first prescription until case assignment**  Median in days (25 - 75%) | **Characteristics of cases** |  | |
|  |  |  |  |  |  |  | |
| **Symptom-defined** |  | 266:183 (68%) |  | 38 (27 – 47) | Liver-related diagnosis  jaundice – symptom  obstructive jaundice nos  [d]jaundice  o/e – jaundiced  [d]jaundice (not of newborn)  yellow/jaundice colour  [d]icterus  o/e – jaundiced colour | N=169  66 (39%)  36 (21%)  27 (16%)  20 (12%)  14 (8%)  3 (2%)  2 (1%)  1 (1%) | |
|  |  |  |  |  | Rash or pruritus | 11 (7%) | |
|  |  |  |  |  | Eosinophilia (n=55)^3^ | 11 (20%) | |
|  |  |  |  |  |  |  | |
|  |  |  |  |  |  |  | |
| **Laboratory-confirmed** |  | 149: 108 (72%) |  | 40 (32 – 48) | Liver-related diagnosis  jaundice – symptom  obstructive jaundice nos  o/e – jaundiced  [d]jaundice  [d]jaundice (not of newborn)  hepatitis unspecified nos  yellow/jaundice colour  toxic liv disease with cholestasis  o/e – jaundiced colour  hepatitis unspecified  hepatitis non a non b  acute hepatitis – noninfective  acute hepatic failure  Type of liver injury  hepatocellular  cholestatic^4^ | N=102  36 (35%)  18 (18%)  14 (14%)  13 (13%)  7 (7%)  4 (4%)  3 (3%)  2 (2%)  1 (1%)  1 (1%)  1 (1%)  1 (1%)  1 (1%)  33 (31%)  75 (69%) | |
|  |  |  |  |  | Rash or pruritus | 7 (7%) | |
|  |  |  |  |  | Eosinophilia (n=48) | 9 (19%) | |
| **Note 1:** Symptom based only: electronic record contains a diagnostic code for any jaundice-related symptom within 90 days of index prescription. Laboratory-confirmed: any of the diagnostic codes listed in supplementary data section 3 present within 90 days of the liver-related diagnosis, and liver test results indicating drug-induced liver injury (according to Aithal ref) present within 90 days either side of this diagnosis (but not before the index prescription). Both definitions: all other more likely causes of the liver symptoms ruled out by clinician review of full electronic health record in the 6-month period before the index prescription. **Note 2:** All within the 90-day period after the index prescription. Potential: people identified as symptom-based or laboratory-confirmed cases of liver injury prior to review of electronic record for other more likely causes. **Note 3:** Eosinophilia classified by an eosinophil count of >0.5 10^9^/l, based upon.[^30^](#_ENREF_30) Only a proportion of all cases had blood eosinophil count measured, indicated by (n=number who were tested). **Note 5:** Of the cholestatic cases, 41 (55%) were pure cholestatic, with the remaining 34 (45%) mixed hepatocellular-cholestatic. | | | | | | |  |

**Table S2: Comparison of multiple imputation analysis with a complete records analysis for variables with missing data in the flucloxacillin (compared with oxytetracycline) and liver injury cohort: crude risk ratios for laboratory-confirmed liver injury**

|  |  |  |  | **Multiple imputation^1^ used to account for missing data** | |  | **Complete records analysis** | |
| --- | --- | --- | --- | --- | --- | --- | --- | --- |
|  |  | **# with outcome** | **People^2^** | **Crude Risk Ratio (CI^3^)** | **p-value^4^** |  | **Crude Risk Ratio (CI)** | **p-value** |
|  |  | n=76 | N=1046699 |  |  |  |  |  |
| **Smoking status** | Non-smoker | 30 | 467185 | 1 | 0.006 |  | 1 | 0.006 |
| (missing n=21755) | Current smoker | 11 | 260101 | 0.64 (0.32 – 1.27) |  |  | 0.66 (0.33 – 1.31) |  |
|  | Ex-smoker | 34 | 297658 | 1.74 (1.08 – 2.85) |  |  | 1.78 (1.09 – 2.91) |  |
|  |  |  |  |  |  |  |  |  |
| **BMI** | <20 | 2 | 59374 | 1 | 0.399 |  | 1 | 0.193 |
| (missing n=141633) | 20 – 25 | 29 | 303273 | 2.33 (0.46 – 11.79) |  |  | 2.84 (0.68 – 11.90) |  |
|  | 25+ | 39 | 542419 | 1.78 (0.34 – 9.18) |  |  | 2.13 (0.52 – 8.84) |  |
|  |  |  |  |  |  |  |  |  |
| **Alcohol intake** | Non-drinker | 4 | 117896 | 1 |  |  | 1 | 0.052 |
| (missing n=146511) | Ex-drinker | 1 | 33858 | 0.78 (0.09 – 7.06) | 0.089 |  | 0.87 (0.10 – 7.70) |  |
|  | Current NOS | 4 | 33304 | 3.51 (0.78 – 15.86) |  |  | 3.54 (0.89 – 14.16) |  |
|  | 2 or less u/d | 22 | 169725 | 3.59 (1.16 – 11.16) |  |  | 3.82 (1.32 – 11.09) |  |
|  | 3/6 u/d | 34 | 465597 | 2.02 (0.70 – 5.80) |  |  | 2.15 (0.76 – 6.07) |  |
|  | >6 u/d | 5 | 79808 | 1.84 (0.51 – 6.68) |  |  | 1.85 (0.50 – 6.88) |  |
|  |  |  |  |  |  |  |  |  |
| **Socioeconomic status** | 1 (Highest SES) | 17 | 186791 | 1 | 0.905 |  | 1 | 0.881 |
| (missing n=235614) | 2 | 14 | 175506 | 0.90 (0.46 – 1.76) |  |  | 0.88 (0.43 – 1.78) |  |
|  | 3 | 14 | 167976 | 0.95 (0.49 – 1.83) |  |  | 0.92 (0.45 – 1.86) |  |
|  | 4 | 12 | 158966 | 0.79 (0.38 – 1.61) |  |  | 0.83 (0.40 – 1.74) |  |
|  | 5 (Lowest SES) | 7 | 121846 | 0.68 (0.24 – 1.90) |  |  | 0.63 (0.26 – 1.52) |  |
|  |  |  |  |  |  |  |  |  |
| **Ethnicity^5^** | White | 46 | 534142 | 1 | 0.402 |  | 1 | 0.998 |
| (missing n=389411) | Other than white | 1 | 17497 | 0.29 (0.03 – 2.57) |  |  | 0.31 (0.04 – 2.23) |  |
|  | Not Stated | 9 | 85336 | 1.27 (0.62 – 2.56) |  |  | 1.22 (0.60 – 2.50) |  |
| **Note 1:** Multiple imputation with chained equations. Imputation model included all variables presented in this table plus age, gender, date of index prescription, outcome status, exposure status and concomitant prescriptions for other causes of liver injury. **Note 2:** Number of people prescribed either flucloxacillin or oxytetracycline. **Note 3:** 95% confidence interval. **Note 4:** p-value results for LRT of an association over all categories of the variable.  **Note 5:** Categories combined due to insufficient numbers of events in original ethnicity categories (shown in Table 2) to allow multiple imputation. | | | | | | | | |

**Table S3: Comparison of multiple imputation with using a complete records approach for variables with missing data in the flucloxacillin (compared with oxytetracycline) and liver injury cohort: crude risk ratios for symptom-defined liver injury**

|  |  |  |  | **Multiple imputation^1^ used to account for missing data** | |  | **Complete records analysis** | |
| --- | --- | --- | --- | --- | --- | --- | --- | --- |
|  |  | **# with outcome** | **People^2^** | **Crude Risk Ratio (CI^3^)** | **p-value^4^** |  | **Crude Risk Ratio (CI)** | **p-value** |
|  |  | n=129 | N=1046699 |  |  |  |  |  |
| **Smoking status** | Non-smoker | 48 | 467137 | 1 | <0.001 |  | 1 | 0.001 |
| (missing n=21755) | Current smoker | 23 | 260078 | 0.85 (0.52 – 1.39) |  |  | 0.86 (0.52 - 1.41) |  |
|  | Ex-smoker | 56 | 297602 | 1.83 (1.25 – 2.69) |  |  | 1.83 (1.25 - 2.69) |  |
|  |  |  |  |  |  |  |  |  |
| **BMI** | <20 | 8 | 59366 | 1 | 0.078 |  | 1 | 0.028 |
| (missing n=141633) | 20 – 25 | 54 | 303219 | 1.26 (0.55 – 2.82) |  |  | 1.32 (0.63 - 2.78) |  |
|  | 25+ | 58 | 542361 | 0.76 (0.36 – 1.62) |  |  | 0.79 (0.38 - 1.66) |  |
|  |  |  |  |  |  |  |  |  |
| **Alcohol intake** | Non-drinker | 11 | 117885 | 1 | 0.054 |  | 1 | 0.069 |
| (missing n=146511) | Ex-drinker | 3 | 33855 | 0.92 (0.27 – 3.14) |  |  | 0.95 (0.26 - 3.40) |  |
|  | Current NOS | 7 | 33297 | 2.11 (0.78 – 5.66) |  |  | 2.25 (0.87 - 5.81) |  |
|  | 2 or less u/d | 34 | 169691 | 2.03 (1.04 – 3.98) |  |  | 2.15 (1.09 - 4.24) |  |
|  | 3/6 u/d | 52 | 465545 | 1.10 (0.56 – 2.14) |  |  | 1.20 (0.62 - 2.29) |  |
|  | >6 u/d | 9 | 79799 | 1.13 (0.48 – 2.66) |  |  | 1.21 (0.50 - 2.92) |  |
|  |  |  |  |  |  |  |  |  |
| **Socioeconomic status** | 1 (Highest SES) | 23 | 186768 | 1 | 0.846 |  | 1 | 0.956 |
| (missing n=235614) | 2 | 22 | 175484 | 1.05 (0.61 – 1.80) |  |  | 1.02 (0.57 - 1.83) |  |
|  | 3 | 23 | 167953 | 1.21 (0.71 – 2.06) |  |  | 1.11 (0.62 - 1.98) |  |
|  | 4 | 17 | 158949 | 0.87 (0.43 – 1.74) |  |  | 0.87 (0.46 - 1.63) |  |
|  | 5 (Lowest SES) | 16 | 121830 | 1.20 (0.66 – 2.19) |  |  | 1.07 (0.56 - 2.02) |  |
|  |  |  |  |  |  |  |  |  |
| **Ethnicity^5^** | White | 78 | 534064 | 1 | 0.282 |  | 1 | 0.065 |
| (missing n=389411) | Other than white | 1 | 17497 | 0.18 (0.02 – 1.55) |  |  | 0.18 (0.03 – 1.30) |  |
|  | Not Stated | 13 | 85232 | 0.97 (0.52 – 1.79) |  |  | 1.04 (0.58 - 1.88) |  |
| **Note 1:** Multiple imputation with chained equations. Imputation model included all variables presented in this table plus age, gender, date of index prescription, outcome status, exposure status and concomitant prescriptions for other causes of liver injury. **Note 2:** Number of people prescribed either flucloxacillin or oxytetracycline. **Note 3:** 95% confidence interval. **Note 4:** p-value results for LRT of an association over all categories of the variable.  **Note 5:** Categories combined due to insufficient numbers of events in original ethnicity categories (shown in Table 2) to allow multiple imputation. | | | | | | | | |

1. Example of calculation of revised numbers needed to treat for an *HLA-B*5701* predictive genetic test (see [^31^](#_ENREF_31) for original calculations)

For the over 70 year olds (incidence of 45/100000):

- Pre-test probability=45/100 000*100=0.045
- Post test probability for a positive test is 0.045*14.5=0.6525% (14.5 is the Likelihood ratio for a positive test)[^31^](#_ENREF_31)
- Post test probability for a negative test is 0.04*0.14=0.0056% (0.14 is the Likelihood ratio for a negative test)[^31^](#_ENREF_31)
- Assuming 7% carrier frequency and a population of 100 000[^31^](#_ENREF_31)
  - 7000 * 0.006525=45.675
  - 93000*0.000063=5.859
- Absolute risk reduction= 45.675-5.859=39.816
- Number of 70 year olds needed to screen to prevent 1 case=100 000/39.816=2512

1. The ten most common diagnostic terms entered on the first day of prescription for either flucloxacillin or oxytetracycline

| Oxytetracycline | | Flucloxacillin | |
| --- | --- | --- | --- |
| Diagnostic term | %^1^ | Diagnostic term | %^1^ |
| Acne vulgaris | 8 | Cellulitis NOS | 5 |
| C/O: a rash | 6 | Skin and subcutaneous tissue infections | 5 |
| Rosacea | 3 | Infected insect bite | 3 |
| C/O - cough | 3 | Impetigo | 3 |
| Acne rosacea | 3 | Infected sebaceous cyst | 3 |
| Cough | 2 | Dressing of wound | 2 |
| Intertrigo | 2 | C/O: a rash | 2 |
| Chest infection NOS | 2 | Folliculitis | 2 |
| Acute sinusitis | 2 | Boil NOS | 2 |
| Chest infection | 2 | Furuncle - boil | 2 |

Note 1: % of people who had this diagnostic code on the date of their first prescription

1. Supplementary Data - References

1. Herrett E, Thomas SL, Schoonen WM et al. Validation and validity of diagnoses in the General Practice Research Database: a systematic review. *British Journal of Clinical Pharmacology* 2010; **69**: 4-14.

2. Campbell J, Dedman DJ, Eaton SC et al. Is the CPRD GOLD Population Comparable to the UK Population? *Pharmacoepidemiology and Drug Safety* 2013; **22**: 280.

3. MHRA. CPRD research papers. <http://www.cprd.com/Bibliography/Researchpapers.asp> (03/01/2014 2014, date last accessed).

4. Cheetham TC, Lee J, Hunt CM et al. An automated causality assessment algorithm to detect drug-induced liver injury in electronic medical record data. *Pharmacoepidemiology and Drug Safety* 2014; **23**: 601-8.

5. De Abajo FJM, D., Madurga M, Garcia Rodriguez LA. Acute and clinically relevant drug-induced liver injury: A population case-control study. *British Journal of Clinical Pharmacology* 2004; **58**: 71-80.

6. García Rodríguez LA, Duque A, Castellsague J et al. A cohort study on the risk of acute liver injury among users of ketoconazole and other antifungal drugs. *British Journal of Clinical Pharmacology* 1999; **48**: 847-52.

7. García Rodríguez LA, Stricker BH, Zimmerman HJ. Risk of acute liver injury associated with the combination of amoxicillin and clavulanic acid. *Archives of Internal Medicine* 1996; **156**: 1327-32.

8. García Rodríguez LA, Wallander MA, Stricker BH. The risk of acute liver injury associated with cimetidine and other acid-suppressing anti-ulcer drugs. *British Journal of Clinical Pharmacology* 1997; **43**: 183-8.

9. García Rodríguez LA, Williams R, Derby LE et al. Acute liver injury associated with nonsteroidal anti-inflammatory drugs and the role of risk factors. *Archives of Internal Medicine* 1994; **154**: 311 - 6.

10. Huerta C, Zhao SZ, García Rodríguez LA. Risk of acute liver injury in patients with diabetes. *Pharmacotherapy* 2002; **22**: 1091-6.

11. Jick H, Derby L, Dean A. Flucloxacillin and cholestatic hepatitis. *The medical journal of Australia* 1994; **160**: 525.

12. Jick H, Derby LE. A large population-based follow-up study of trimethoprim-sulfamethoxazole, trimethoprim, and cephalexin for uncommon serious drug toxicity. *Pharmacotherapy* 1995; **15**: 428-32.

13. Li L, Jick H, Jick SS. Updated study on risk of cholestatic liver disease and flucloxacillin: Letter to the Editors. *British Journal of Clinical Pharmacology* 2009; **68**: 269-70.

14. Russmann S, Kaye JA, Jick SS et al. Risk of cholestatic liver disease associated with flucloxacillin and flucloxacillin prescribing habits in the UK: Cohort study using data from the UK General Practice Research Database. *British Journal of Clinical Pharmacology* 2005; **60**: 76-82.

15. Shin J, Hunt CM, Suzuki A et al. Characterizing phenotypes and outcomes of drug-associated liver injury using electronic medical record data. *Pharmacoepidemiol Drug Saf* 2013; **22**: 190-8.

16. Wing K, Bhaskaran K, Smeeth L et al. Optimising case detection within UK electronic health records: use of multiple linked databases for detecting liver injury. *BMJ Open* 2016; **6**.

17. Danan G, Benichou C. Causality assessment of adverse reactions to drugs--I. A novel method based on the conclusions of international consensus meetings: application to drug-induced liver injuries. *J Clin Epidemiol* 1993; **46**: 1323-30.

18. Greenland S, Pearl J, Robins J. Causal Diagrams for Epidemiological Research. *Epidemiology* 1999; **10**: 37-48.

19. Textor J, Hardt J, Knüppel S. DAGitty: A Graphical Tool for Analyzing Causal Diagrams. *Epidemiology* 2011; **22**: 745 10.1097/EDE.0b013e318225c2be.

20. Derby LE, Jick H, Henry DA et al. Cholestatic hepatitis associated with flucloxacillin. *Medical Journal of Australia* 1993; **158**: 596-600.

21. Fairley CK, McNeil JJ, Desmond P et al. Risk factors for development of flucloxacillin associated jaundice. *Bmj* 1993; **306**: 233-5.

22. Daly AK. Drug-induced liver injury: past, present and future. *Pharmacogenomics* 2010; **11**: 607-11.

23. Daly AK, Donaldson PT, Bhatnagar P et al. HLA-B[ast]5701 genotype is a major determinant of drug-induced liver injury due to flucloxacillin. *Nat Genet* 2009; **41**: 816-9.

24. Mathur R, Bhaskaran K, Chaturvedi N et al. Completeness and usability of ethnicity data in UK-based primary care and hospital databases. *Journal of Public Health* 2013.

25. Padda MS, Sanchez M, Akhtar AJ et al. Drug-induced cholestasis. *Hepatology* 2011; **53**: 1377-87.

26. Suzuki A, Andrade R, Bjornsson E et al. Drugs Associated with Hepatotoxicity and their Reporting Frequency of Liver Adverse Events in VigiBase™. *Drug-Safety* 2010; **33**: 503-22.

27. Velayudham LS, Farrell GC. Drug-induced cholestasis. *Expert Opinion on Drug Safety* 2003; **2**: 287-304.

28. NIH. Agents Included in LiverTox by Drug Class. <http://livertox.nih.gov/drugliverinjury.html> (01/05/2014 2014, date last accessed).

29. Sterne JAC, White IR, Carlin JB et al. Multiple imputation for missing data in epidemiological and clinical research: potential and pitfalls. *BMJ* 2009; **338**.

30. Butt NM, Lambert J, Ali S et al. Guideline for the investigation and management of eosinophilia. *British Journal of Haematology* 2017; **176**: 553-72.

31. Alfirevic A, Pirmohamed M. Predictive genetic testing for drug-induced liver injury: considerations of clinical utility. *Clin Pharmacol Ther* 2012; **92**: 376-80.
